# RIP-seq reveals RNAs that interact with RNA polymerase and primary sigma factors in bacteria

**DOI:** 10.1093/nar/gkae081

**Published:** 2024-02-13

**Authors:** Viola Vaňková Hausnerová, Mahmoud Shoman, Dilip Kumar, Marek Schwarz, Martin Modrák, Jitka Jirát Matějčková, Eliška Mikesková, Silvia Neva, Anna Herrmannová, Michaela Šiková, Petr Halada, Iva Novotná, Petr Pajer, Leoš Shivaya Valášek, Martin Převorovský, Libor Krásný, Jarmila Hnilicová

**Affiliations:** Laboratory of Microbial Genetics and Gene Expression, Institute of Microbiology of the Czech Academy of Sciences, Prague142 20, Czech Republic; Laboratory of Regulatory RNAs, Faculty of Science, Charles University, Prague128 44, Czech Republic; Laboratory of Microbial Genetics and Gene Expression, Institute of Microbiology of the Czech Academy of Sciences, Prague142 20, Czech Republic; Laboratory of Regulatory RNAs, Faculty of Science, Charles University, Prague128 44, Czech Republic; Laboratory of Microbial Genetics and Gene Expression, Institute of Microbiology of the Czech Academy of Sciences, Prague142 20, Czech Republic; Laboratory of Bioinformatics, Institute of Microbiology of the Czech Academy of Sciences, Prague142 20, Czech Republic; Laboratory of Bioinformatics, Institute of Microbiology of the Czech Academy of Sciences, Prague142 20, Czech Republic; Department of Bioinformatics, Second Faculty of Medicine, Charles University, Prague150 06, Czech Republic; Laboratory of Microbial Genetics and Gene Expression, Institute of Microbiology of the Czech Academy of Sciences, Prague142 20, Czech Republic; Laboratory of Regulatory RNAs, Faculty of Science, Charles University, Prague128 44, Czech Republic; Laboratory of Microbial Genetics and Gene Expression, Institute of Microbiology of the Czech Academy of Sciences, Prague142 20, Czech Republic; Laboratory of Regulatory RNAs, Faculty of Science, Charles University, Prague128 44, Czech Republic; Laboratory of Microbial Genetics and Gene Expression, Institute of Microbiology of the Czech Academy of Sciences, Prague142 20, Czech Republic; Laboratory of Regulatory RNAs, Faculty of Science, Charles University, Prague128 44, Czech Republic; Laboratory of Regulation of Gene Expression, Institute of Microbiology of the Czech Academy of Sciences, Prague142 20, Czech Republic; Laboratory of Microbial Genetics and Gene Expression, Institute of Microbiology of the Czech Academy of Sciences, Prague142 20, Czech Republic; Laboratory of Structural Biology and Cell Signaling, Institute of Microbiology of the Czech Academy of Sciences, Vestec252 50, Czech Republic; Military Health Institute, Military Medical Agency, Prague169 02, Czech Republic; Military Health Institute, Military Medical Agency, Prague169 02, Czech Republic; Laboratory of Regulation of Gene Expression, Institute of Microbiology of the Czech Academy of Sciences, Prague142 20, Czech Republic; Department of Cell Biology, Faculty of Science, Charles University, Prague128 00, Czech Republic; Laboratory of Microbial Genetics and Gene Expression, Institute of Microbiology of the Czech Academy of Sciences, Prague142 20, Czech Republic; Laboratory of Microbial Genetics and Gene Expression, Institute of Microbiology of the Czech Academy of Sciences, Prague142 20, Czech Republic; Laboratory of Regulatory RNAs, Faculty of Science, Charles University, Prague128 44, Czech Republic

## Abstract

Bacteria have evolved structured RNAs that can associate with RNA polymerase (RNAP). Two of them have been known so far—6S RNA and Ms1 RNA but it is unclear if any other types of RNAs binding to RNAP exist in bacteria. To identify all RNAs interacting with RNAP and the primary σ factors, we have established and performed native RIP-seq in *Bacillus subtilis, Corynebacterium glutamicum*, *Streptomyces coelicolor*, *Mycobacterium smegmatis* and the pathogenic *Mycobacterium tuberculosis*. Besides known 6S RNAs in *B. subtilis* and Ms1 in *M. smegmatis*, we detected MTS2823, a homologue of Ms1, on RNAP in *M. tuberculosis*. In *C. glutamicum*, we discovered novel types of structured RNAs that associate with RNAP. Furthermore, we identified other species-specific RNAs including full-length mRNAs, revealing a previously unknown landscape of RNAs interacting with the bacterial transcription machinery.

## Graphical Abstract

**Graphical Abstract:**
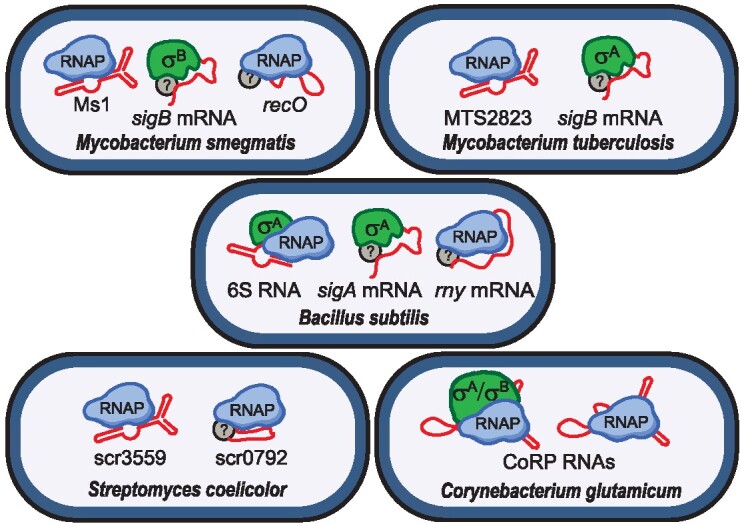

## Introduction

Bacteria are the most abundant and diverse group of organisms on earth. Certain bacteria are life-threatening human pathogens, such as *Mycobacterium tuberculosis* causing tuberculosis, an infectious disease that affects over 10 million people every year (https://www.who.int/teams/global-tuberculosis-programme/tb-reports/global-tuberculosis-report-2022).

Bacterial transcription is an important target of antibiotics. Rifampicin, which inhibits bacterial RNA polymerase (RNAP), is still the first-line drug to treat tuberculosis, although it was discovered >50 years ago (1). The RNAP core consists of several subunits (α_2_ββ′ω, ∼400 kDa) and together with a σ factor forms the RNAP holoenzyme (2). σ factors are necessary to initiate transcription. All bacteria have one primary or housekeeping σ factor [σ^70^ in *Escherichia coli*, σ^A^ in *Bacillus subtilis* or *Mycobacterium smegmatis* (3) or HrdB in *Streptomycetes coelicolor* (4)] that recognizes promoters of essential genes required for exponential growth, and various numbers of alternative σ factors that are involved in transcription of genes during stress conditions (5).

**Figure 1.**
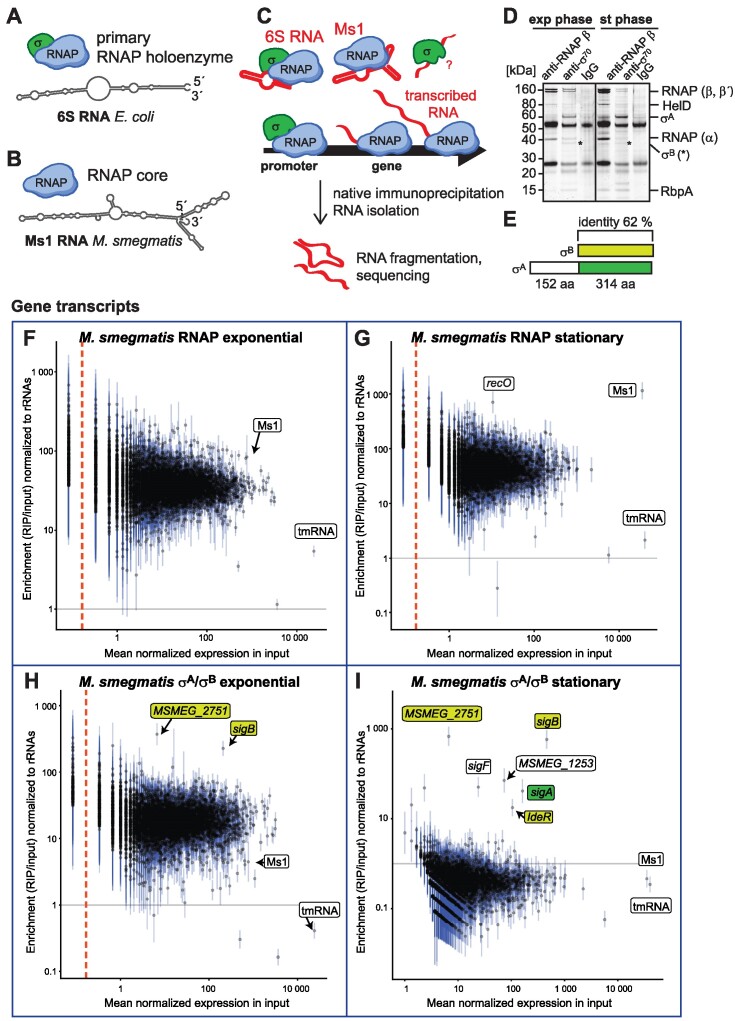
RIP-seq in *Mycobacterium smegmatis*. (**A**) The primary RNAP holoenzyme consists of RNAP core with the primary σ factor and associates with 6S RNA. The secondary structure of 6S RNA from *E. coli* is shown. (**B**) Ms1 (predicted secondary structure from *M. smegmatis*) binds to core RNAP lacking a σ factor. (**C**) RIP-seq detects both RNA molecules binding to RNAPs or σ factors or RNAP-σ holoenzymes and also nascent RNAs that are connected with the transcribing RNAP-σ holoenzymes or RNAPs. (**D**) The immunoprecipitated proteins visualized by silver-stained SDS-PAGE. The anti-RNAP antibody that recognizes the β subunit precipitates mainly the core RNAP and additional RNAP-associated proteins (HelD, *MSMEG_2174* (52) and RbpA, *MSMEG_3858* (98,99)). The anti-σ^70^ antibody binds both σ^A^ and σ^B^ and also their RNAP holoenzyme complexes, because of very similar protein sequences (**E**). (**F**–**I**) Quantification of RIP-seq data in *M. smegmatis* in exponential and stationary phase for RNAP (**F, G**) and σ^A^/σ^B^ (**H, I**) for each annotated gene. For intergenic regions, please see Supplementary Figure S2. The horizontal axis (log scale) represents the mean of the normalized counts in the input, vertical axis (log scale) shows the estimated fold change in the ratio of read counts in immunoprecipitated to input samples, each normalized to the read counts for rRNA. Estimates (points) and 95% confidence intervals (lines) are shown for all transcripts. Transcripts to the left of the vertical dashed line had zero reads in all replicates of the input material. The horizontal gray line marks a fold change of 1, i.e. no enrichment or depletion of the respective RNA transcript after immunoprecipitation.

The primary σ-RNAP holoenzyme interacts with 6S RNA (Figure 1A) (6,7), a structured, non-coding RNA that resembles an open promoter (8,9). 6S RNA has been first identified and mostly studied in the gram-negative model organism *E. coli* (10–17), where it accumulates in the stationary phase of growth and sequesters the primary σ-RNAP holoenzyme (9,18). Similar to the promoter DNA during transcription initiation, 6S RNA binds to the σ-RNAP holoenzyme (17–19). In *E. coli*, the majority of σ^70^-RNAP holoenzyme is associated with 6S RNA in late stationary phase (8,9) and the interaction of σ^70^-RNAP with 6S RNA results in global changes in gene expression (12,14).

6S RNA can serve as a template for σ-RNAP to transcribe product RNAs (pRNAs), usually <20 nt in length (7,20–23). Longer pRNAs (about ≥13 nt), which remain stably bound to 6S RNA, induce persistent structural rearrangements in 6S RNA, thereby reducing 6S RNA affinity for σ-RNAP, which consequently leads to the release of σ and RNAP (21,24–26). The overall synthesis of pRNAs and the fraction of longer length species (≥13 nt) increase during outgrowth from stationary phase (21,27), likely due to elevated NTP concentrations. Subsequently, the released σ and RNAP can immediately resume transcription at DNA promoters.

Although the primary nucleotide sequence of 6S RNA is not conserved, its length (∼180 nt) and the secondary structure are conserved among different bacterial species (8,28). Therefore, many 6S RNA genes have been identified based on bioinformatic RNA structure searches (8,28,29). However, in the actinobacteria group, which includes mycobacteria and corynebacteria, 6S RNA has not been identified yet, except for one 6S RNA candidate in *Streptomyces coelicolor* (30,31).

Previously, we identified Ms1 RNA (32) which is highly abundant in stationary phase in *Mycobacterium smegmatis* and associates with the RNAP core without the primary σ factor, σ^A^ (Figure 1B) (33). Ms1 regulates the amount of RNAP in stationary phase (34) and helps mycobacteria accelerate outgrowth in rich medium (34). Although *Mycobacterium tuberculosis* has an Ms1 homolog (MTS2823 RNA) (35), it has never been determined whether MTS2823 associates with RNAP in *M. tuberculosis*. It is currently unclear if any 6S RNA is present in mycobacteria or if any other RNA interacts with σ^A^.

Recently, putative Ms1 RNAs homologs have been found among many actinobacteria, for example in *Streptomyces coelicolor* (36,37). In some actinobacteria, such as corynebacteria, neither 6S RNA nor Ms1 have been discovered so far (37) raising the question whether any similar RNA exists in these species. It is also unknown if both Ms1 and 6S RNA are present in *Streptomyces coelicolor*.

To identify a complete set of RNAs that associate with the bacterial transcription machinery, we have established a native RIP-seq protocol and applied it to five bacterial species – *Mycobacterium smegmatis* and the pathogenic *Mycobacterium tuberculosis*, the producers of antibiotics and amino acids – *Streptomyces coelicolor* and *Corynebacterium glutamicum*, respectively, and the model gram-positive bacterium *Bacillus subtilis*. We have identified RNAs that were specifically enriched either on RNAP or on the primary σ factor. These RNAs can be divided into three groups – abundant RNAP-associating RNAs (such as 6S RNA, Ms1 and newly identified CoRP in corynebacteria); full-length mRNAs that are functionally linked to RNA transcription or degradation; and sRNAs or mRNA fragments that are often low expressed, but still significantly associate with RNAP. Bacteria thus contain diverse sets of ribonucleoprotein complexes that are involved in bacterial transcription.

## Materials and methods

### Bacterial strains, growth conditions

*Mycobacterium smegmatis* mc^2^ 155 cells (*wt*, ATCC no. 700084, *LK865*) were grown at 37°C in Middlebrook 7H9 medium with 0.2% glycerol, 0.05% Tween 80 and then harvested in exponential (OD_600_ ∼0.5) and early stationary phase (OD_600_ ∼2.5–3, 24 h of cultivation). For mitomycin C treatment, the antibiotic was added at OD_600_ ∼0.5 to a final concentration of 80 ng/ml, followed by cell cultivation for 4 h. For the heat shock, bacteria were transferred to 45°C after reaching an OD_600_ of ∼0.5 and cultivated for 4 h. For the osmotic shock, after reaching OD_600_ ∼0.5, NaCl was added to the medium at a final concentration of 0.5 M and bacteria were cultivated for 4 h. *Mycobacterium tuberculosis* H37Rv (ATCC no. 27294) cells were first grown on Löwenstein–Jensen agar plates for 21 days and then inoculated into Middlebrook 7H9 medium supplemented with 10% (v/v) OADC, 0.2% glycerol and 0.05% Tween 80 and cultivated at 35.5°C for 15 days which corresponds to early stationary phase. *Streptomyces coelicolor A3(2)* spore stock expressing HA-tagged HrdB (38) was thawed to inoculate 2x YT medium. Germination was carried out at 30°C for 5 h as previously described (38,39). The germinated spores were harvested, inoculated into Na-glutamate medium supplemented with trace element solution and TMS1 as previously described (38), cultivated at 30°C and harvested at different time points after germination (42 h, ∼exponential phase, 66 h, ∼stationary phase). *Corynebacterium glutamicum* cells (wt, ATCC no. 13032, *LK1100*) were grown at 30°C in 2x YT medium and harvested in exponential (OD_600_ ∼ 1) or early stationary phase (OD_600_ > 7, ∼26 h of cultivation). *Bacillus subtilis* 168 trp+ (BaSysBio cells, *LK2711*) and *Escherichia coli* strains (K12KW72, *LK1133*) (15) were inoculated into LB medium at OD_600_ 0.03 and grown at 37°C. Bacteria were harvested in exponential (OD_600_ ∼0.25–0.4) or stationary phase (OD_600_ ∼3–4, 7–8 h of cultivation).

### RIP-seq, RNA immunoprecipitation, RT-qPCR and RT-PCR

#### Immunoprecipitation

Cells were pelleted and washed in lysis buffer [20 mM Tris–HCl pH 7.9, 150 mM KCl, 1 mM MgCl_2_], pelleted again and these pellets were frozen at -70°C. Bacterial pellets were resuspended in lysis buffer supplemented with phenylmethylsulfonyl fluoride (PMSF) and Protease inhibitor cocktail [20 mM Tris–HCl pH 7.9, 150 mM KCl, 1 mM MgCl_2_, 1 mM dithiothreitol (DTT), 0.5 mM PMSF, Protease Inhibitor Cocktail Set III protease inhibitors (Calbiochem)], sonicated 15 × 10 s with 1 min pauses on ice and centrifuged at 8960 × g for 15 min at 4°C. For *M. tuberculosis*, cells were resuspended in lysis buffer supplemented with PMSF and Protease inhibitors cocktail [20 mM Tris–HCl pH 7.9, 150 mM KCl, 1 mM MgCl_2_, 1 mM DTT), 0.5 mM PMSF, Calbiochem Protease Inhibitor Cocktail Set III protease inhibitors] and disrupted by vortexing with 0.1 mm zirconia beads 7 × 30 s with 1 min pauses on ice and centrifuged. 0.5–4 mg of protein lysates were incubated for 16–18 h (for *M. tuberculosis*, for 4 h) at 4°C with 20 μl of Protein G plus agarose beads (Santa Cruz Biotechnology) coated with 5 μg of anti- RNAP beta subunit antibody [clone 8RB13] (BioLegend), 2.5 μg of anti- σ^70^ antibody [clone 2G10] (BioLegend), or 5 μg of non-specific IgG (Sigma-Aldrich, Cat. No. I5381) used as a negative control, respectively.

The captured complexes were washed four times using 20 mM Tris–HCl pH 7.9, 150 mM KCl, 1 mM MgCl_2_ and finally resuspended in 300 μl. 2/3 of the immunoprecipitated sample were used for RNA isolation, which involved incubation on a rotating platform with 200 μl acidic phenol (pH ∼ 3)/chloroform (1:1) and 0.5% SDS for 15 min. Eluted RNA was precipitated with ethanol and dissolved in 20 μl double distilled water and treated with DNase (TURBO DNA-free Kit, Ambion). For inputs, 10% of lysate that was used for one immunoprecipitation was diluted in 20 mM Tris–HCl pH 7.9, 150 mM KCl, 1 mM MgCl_2_ to final volume of 200 μl and RNA was isolated with the same protocol as the immunoprecipitated RNA. The remaining 1/3 of the immunoprecipitated sample was mixed with 4x SDS sample buffer (200 mM Tris–Cl (pH 6.8), 8% SDS, 0.4% Bromophenol blue and 40% glycerol), heated at 95°C for 5 min and resolved on SDS-PAGE gels (see the section SDS-PAGE, western blotting).

#### Library preparation and sequencing

14 μl of RNA sample were used for library construction according to the NEXTFLEX® Rapid Directional RNA-Seq Kit. For input samples, 100 ng of RNA was used for library construction. RNA in control IgG libraries was not detectable, therefore these libraries were not sequenced. Pooled barcoded libraries were sequenced in single lanes using the Illumina NextSeq® 500/550 High Output Kit v2 in 75 bp single end regime at the Institute of Molecular Genetics AS CR, Prague, Czech Republic.

#### Reverse transcription and RT-qPCR

5 μl RNA was reverse transcribed into cDNA (20 μl reaction, SuperScriptIII, Invitrogen) using random hexamers and amplified by RT-qPCR in a LightCycler 480 System (Roche Applied Science) in duplicate reactions containing LightCycler 480 SYBR Green I Master and 0.5 μM primers (each). Primers were designed with Primer3, sequences are listed in the Supplementary Data. Negative controls (no template reactions and reactions with RNA as a template to control for contamination with genomic DNA) were run in each experiment, the quality of the PCR products was determined by dissociation curve analysis and the efficiency of the primers determined by standard curves. The relative amounts of co-immunoprecipitated RNAs were quantified on the basis of threshold cycles (Ct) for each PCR product that was normalized to input values according to the formula 2(Ct^(immunoprec)^ − Ct^(input)^). For RT-PCR, 1:10 diluted cDNA was amplified by Biotools DNA polymerase in 25 μl reaction (30 cycles). For no RT controls, RNA was diluted 1:40 and added to the qPCR reaction to ensure that no gDNA contamination was present.

### ChIP-seq

100 ml of bacterial culture was crosslinked with 1% formaldehyde (final concentration in the medium) for 30 min at 37°C in the shaker. Formaldehyde was quenched by glycine (0.125 M final concentration) added for 5 min at 37°C in the shaker. Bacterial cultures were centrifuged for 5 min at 8960 × g, 4°C, the pellet washed with 10 ml of 1× PBS, bacteria centrifuged again and the pellet immediately frozen at –70°C. Bacterial pellet was resuspended in 3 ml of ice cold RIPA buffer (150 mM NaCl, 1% Triton X-100, 0.5% deoxycholate, 0.1% SDS, 50 mM Tris–HCl pH 8.0, 5 mM EDTA) with protease inhibitors cocktail (Sigma-Aldrich) and 0.5 mM PMSF (5 μl/ml) and sonicated 18 × 10 s with 1 min pauses on ice between cycles. The lysate was centrifuged at 8960 × g and 4°C for 15 min, the pellet was discarded. A volume of supernatant corresponding to 2 mg of protein was incubated for 16–18 h at 4°C with 20 μl of Protein G plus agarose beads (Santa Cruz Biotechnology) coupled with 5 μg anti-RNAP β subunit antibody [clone 8RB13] (BioLegend), 2.5 μg of anti-σ^70^ antibody [clone 2G10] (BioLegend), or 5 μg of non-specific IgG (Sigma-Aldrich, Cat. No. I5381) used as a negative control, respectively. The captured complexes were washed twice with RIPA buffer (150 mM NaCl, 1% Triton X-100, 0.5% deoxycholate, 0.1% SDS, 50 mM Tris–HCl pH 8.0, 0.5 mM EDTA), four times with LiCl buffer (100 mM Tris–HCl, pH 8.5, 500 mM LiCl, 1% Triton X-100, 1% deoxycholate), two times with RIPA and twice with TE buffer (10 mM Tris–HCl pH 8.0, 1 mM EDTA). Protein-DNA complexes were eluted with elution buffer (50 mM Tris–HCl pH 8, 0.66 mM EDTA, 1% SDS) for 10 min at 65°C, decrosslinked in the presence of 200 mM NaCl for 5 h at 65°C, treated with 100 μg/ml RNase A for 1 h at 37°C and 400 μg/ml proteinase K for 30 min at 45°C. DNA was purified with the QIAGEN PCR purification kit and eluted with 100 μl of Elution Buffer. 40 μl of immunoprecipitated DNA sample or 10 ng of DNA input were used for library construction according to the NEXTFLEX® ChIP-Seq Kit manual including the Size-Selection Cleanup step B2. Pooled barcoded libraries (one biological triplicate) were sequenced in single lanes using the Illumina NextSeq® 500/550 High Output Kit v2 in 75 bp single end regime.

### NGS data processing and analysis

Read quality was checked using FastQC version 0.11.9 (https://www.bioinformatics.babraham.ac.uk/projects/fastqc/). When needed, adapters and low-quality sequences were removed using Trimmomatic 0.39 (40). Reads were aligned to the reference genome using HISAT2 2.2.1 (41) and SAMtools 1.9 (42,43). Read coverage tracks were computed using deepTools 3.5.1 (44). For ChIP-seq, peak calling was done with MACS2 (45) on each replicate separately and only the peak regions overlapping in all replicates were retained as resulting final peaks. The highest (worst) *P*-value from the overlapping peaks was assigned to the resulting peak as its *P*-value. The nature of the RIP-seq experiment means that the input and the IP samples will have vastly different total abundances of RNA and thus the compositional nature of the sequencing data cannot be ignored. Instead of directly considering differences in transcript abundance, we thus focus on differences in ratios of two transcripts, which are meaningful even with vastly different absolute abundances (46, https://doi.org/10.1101/564955). We primarily used the sum of ribosomal RNA reads as denominator/reference. We used the DESeq2 R package (47), for this analysis, *P*-values were corrected for multiple comparisons using the Benjamini-Hochberg method (48) to control the false-discovery rate (FDR) at 5% level. To choose the candidates for RNAP or primary sigma factors interacting RNAs, we have used the following criteria: i. the RNA was characterized by a *P*-value ranked among the top 10 lowest values in the particular dataset, ii. it had a detectable expression level in the input, iii. and/or it was of other interest to us, such Ms1 in *M. smegmatis* and its candidates in the other organisms or 6S RNAs in *B. subtilis*. The code has been deposited at Zenodo under DOI: 10.5281/zenodo.10286942.

### RNA secondary structure analysis

To detect stable RNA secondary structure, the *MSMEG_2752* and *Rv2710* regions of *M. smegmatis* and *M. tuberculosis*, respectively, were analyzed by RNALfold (49) and the number of locally stable structures (RNALfold -z -L 400) at each position of a region was plotted. The investigated regions were defined as 50 bp upstream to 150 bp downstream of each gene. To predict the minimum free energy (MFE) secondary structures of *MSMEG_2752 (sigB)* and *MSMEG_4491 (recO)* of *M. smegmatis* and the scr0792 intergenic region of *S. coelicolor*, the sequence of each transcript or fragment thereof which was enriched in the RIP-seq experiments with anti-RNAP antibody (*recO*, scr0792) or anti-σ^70^ antibody (*sigB*) was submitted to the RNAfold webserver (50,51) (http://rna.tbi.univie.ac.at/cgi-bin/RNAWebSuite/RNAfold.cgi). Additionally, the locally stable secondary structures within the predicted MFE structures were identified based on the RNALfold analysis of each RIP-seq enriched sequence (RNALfold -z –noLP). The code has been deposited at Zenodo under DOI: 10.5281/zenodo.10286942.

### SDS-PAGE, western blotting

Immunoprecipitated proteins were resolved by SDS-PAGE (Nu-PAGE, 4–12% Bis–Tris precast gels, Invitrogen) and stained with Coomassie or silver-stained using the Pierce Silver Stain kit for Mass Spectrometry (Thermo Fisher Scientific). Proteins were detected by western blotting using an anti-RNAP β subunit antibody [clone 8RB13] (BioLegend) or anti-σ^70^ antibody [clone 2G10] (BioLegend), and a HRP-labeled anti-mouse IgG antibody (Sigma-Aldrich). The identity of the protein bands was determined by MALDI-TOF mass spectrometry as described previously (52).

### RNA isolation and northern blotting, RACE

Before total RNA extraction a Plat mRNA spike-in (718 bp) from *M. musculus* was added (Plat mRNA was prepared by *in vitro* transcription from pJET_Plat_IVTs plasmid using the MEGAscript T7 Transcription Kit [Thermo Fisher Scientific], the sequence of Plat mRNA is provided in the Supplementary Data). The amount of RNA spike-in was 1.8 ng per 30 ml of culture at an OD_600_ of 0.5. Each frozen cell pellet was resuspended in 240 μl TE buffer (pH 8.0) plus 60 μl LETS buffer (50 mM Tris–HCl pH 8.0, 500 mM LiCl, 50 mM EDTA pH 8.0, 5% SDS) and 600 μl acidic (pH∼3) phenol/chloroform (1:1). Lysates were sonicated in a fume hood, centrifuged, the aqueous phase extracted two more times with acidic phenol/chloroform and precipitated with ethanol. RNA was dissolved in double distilled water and treated with DNase (TURBO DNA-free Kit, Ambion). 5 μl RNA (∼2.5 μg) was reverse transcribed into cDNA (20 μl reaction, SuperScriptIII, Invitrogen) using random hexamers and amplified by RT-qPCR in a LightCycler 480 System (Roche Applied Science). The mRNA level was normalized to the value of the Plat mRNA spike-in according to the formula 2^(Ct^(spike)^ − Ct^(mRNA)^) and expression (E) normalized to the nontreated strain (*E* = *E*_treatment_/*E*_control_).

RNAs were resolved on a 7 M urea 7% polyacrylamide gel and transferred onto an Amersham Hybond-N membrane or Zeta-Probe nylon membrane (Biorad) according to the protocol described in Panek et al. (32). 5′ biotinylated oligonucleotide probes (Supplementary Data) were hybridized to the membrane and detected with the BrightStar BioDetect Kit (Ambion) or Novex or Tropix CDP STAR substrate (ThermoFischer Scientific, Applied Biosystems) according to the manufacturer's instructions.

5′ RACE and 3′ RACE were performed according to the protocol used previously (37).

### Plasmid construction and *in vitro* transcription

The plasmid utilized as template for transcribing CoRP RNA *in vitro* was prepared in the following way: *C. glutamicum* genomic DNA served as a PCR template and primers Cg_CoRP_iv_F (primer containing the T7 promotor as 5′ overhang) and Cg_CoRP_iv_R (primer containing the bait sequence 5′GGGAGACCTAGCCT 3′) were used. The amplified CoRP sequence was cloned into pUC18 plasmid via *Hin*dIII and *Xba*I restriction sites. Resulting constructs were transformed into *E. coli* DH5α (LK4053) and verified by sequencing.

CoRP templates were linearized with *Xba*I. CoRP RNA was prepared with a T7 RiboMAX Express Large Scale RNA Production System (Promega) according to the manufacturer's instructions.

### Protein pull-down via *in vitro* transcribed RNAs

For protein pull-down using an RNA bait sequence, a modified version of a published protocol (53) was used. For the pull-down, 50 μl of magnetic streptavidin beads (Dynabeads M-280, Thermo Fisher Scientific) were washed 3 times with 1 ml of lysis buffer B (50 mM Tris–HCl, pH 8, 150 mM KCl, 1 mM MgCl_2_, 5% glycerol). The rest of the protocol was performed at 4°C. The washed beads were coupled to 8 μg of a 3′-O-biotinylated, 2′-O-methyl modified RNA adaptor complementary to the 14 nt tag of the bait RNA (AGGCUAGGUCUCCC-biotin) for 1 h. The adaptor-coupled beads were washed twice with 1 ml of lysis buffer B and resuspended in 200 μl of lysis buffer B. The beads were coupled with 10 μg of the *in vitro* prepared CoRP RNA tagged with the bait sequence overnight. The beads for negative control were coupled just with the bait adaptor. For lysate pre-clearing, 50 μl magnetic streptavidin-coated beads (Sigma-Aldrich) were prepared by the same protocol as for the pull-down. The lysate for the pull-down was prepared in the same way as for the RIP-seq experiments. 35 mg and 50 mg of cell extract from exponential and stationary phase, respectively, were incubated with adaptor-coated beads to pre-clear the lysate for 3.5 h.

The bait RNA-coupled beads for the pull-down were washed twice with lysis buffer B and incubated with the pre-cleared lysate supernatant for 2 h to capture proteins interacting with the bait RNAs. The beads were subsequently washed with 1 ml of washing buffer A (lysis buffer B with 300 mM KCl) and 3 times with lysis buffer B. The beads were resuspended in SDS sample buffer, boiled for 5 min at 95°C and the eluted proteins were resolved on SDS-PAGE and detected by Coomassie staining and by silver staining (Pierce Silver Stain kit for Mass Spectrometry, ThermoFisher Scientific). The identity of the bands was confirmed by western blotting.

### Glycerol gradient ultracentrifugation

*M. smegmatis* and *C. glutamicum* stationary phase cells were pelleted and resuspended in 20 mM Tris–HCl pH 8, 150 mM KCl, 1 mM MgCl_2_, 1 mM dithiothreitol (DTT), 0.5 mM phenylmethylsulfonyl fluoride (PMSF) and Calbiochem Protease Inhibitor Cocktail Set III protease inhibitors, sonicated 15 × 10 s with 1 min pauses on ice and centrifuged. Protein extracts (1 mg) were loaded on a linear 10–30% glycerol gradient prepared in gradient buffer (20 mM Tris–HCl pH 8, 150 mM KCl, 1 mM MgCl_2_) and fractionated by centrifugation at 32 000 rpm (130 000 × g) for 17 h using an SW-41 rotor (Beckman). The gradient was divided into 20 (for *M. smegmatis*) or 19 (for *C. glutamicum*) fractions, RNA from individual fractions was extracted with acidic phenol (pH∼3):chloroform, precipitated by ethanol and used for northern blotting and/or RT-qPCR. Proteins were analyzed on SDS-PAGE and detected by western blotting.

### Trypsin digestion and LC–MS/MS analysis

The immunoprecipitated proteins were resuspended in 100 μl of 2 M urea/50 mM Tris–HCl/5 mM DTT and reduced at ambient temperature for a 30 min. After the addition of iodoacetamide (20 mM final concentration), the samples were incubated for another 30 min. The proteins were digested using trypsin (400 ng; Promega) at 37 °C overnight. After digestion, samples were acidified with formic acid (FA) at a final concentration of 1%, desalted on a peptide microtrap (Optimize Technologies) according to the manufacturer instructions and dried.

LC–MS/MS analyses were performed using a Vanquish Neo UHPLC system (Thermo Fisher Scientific) coupled to a timsTOF SCP mass spectrometer (Bruker Daltonics). The dried peptides were dissolved in 25 μl of double-distilled water containing 2% acetonitrile (ACN) and 0.1% FA and separated at a flow rate of 1.5 μl/min on an analytical column (PepSep C18, 0.15 × 150 mm, 1.5 μm; Bruker Daltonics) heated to 50°C using a 45 min-long linear gradient from 100% phase A (0.1% FA) to a 35% phase B (80% ACN/0.1% FA). The MS acquisition method involved recording spectra within the mass range of 100–1700 m/z with ion mobility scanning from 0.6 to 1.6 V s/cm^2^. The method consisted of a TIMS survey scan of 166 ms followed by 10 PASEF MS/MS scans. The total cycle time was 1.9 s. Precursors for data-dependent acquisition were fragmented with an ion mobility-dependent collision energy that increased linearly from 20 to 59 eV.

The raw data were processed using DataAnalysis 5.3 (Bruker Daltonics) and the generated MGF files were searched against UniProt 2023_10 subsets of *M. smegmatis* strain mc^2^155 or *C. glutamicum* strain ATCC 13032 using an in-house MASCOT v.2.7 (Matrix Science) search engine with the following settings: precursor mass tolerance 10 ppm, fragment ion tolerance 0.2 Da, enzyme trypsin/P, missed cleavages set to 2, variable oxidation of methionine and carbamidomethylation of cysteine. The proteins with at least two matching unique peptide sequences were considered as reliably identified.

## Results

### Novel σ^A^-associated RNAs in *Mycobacterium smegmatis*

First, we focused on RNAs associated with the transcriptional machinery in *Mycobacterium smegmatis*. We showed previously that Ms1 RNA binds to the RNAP core (RNAP without σ factor) in stationary phase (Figure 1B) (33). No RNA interacting with σ^A^ has been discovered so far in this species. Furthermore, it is still not fully resolved whether 6S RNA is present in mycobacteria.

We immunoprecipitated RNAP and the primary σ factor (σ^A^) from exponential and stationary phase with anti-RNAP β and anti-σ^70^ specific antibodies, respectively (Figure 1C and D). The anti-σ^70^ antibody immunoprecipitated also σ^B^ since it has a very similar amino acid sequence in mycobacteria (Figure 1E and Supplementary Figure S1). σ^A^ has an additional N-terminal domain of unknown function that is not present in σ^B^, but both σ^A^ and σ^B^ are able to recognize the same -10 and -35 promoter elements (54,55). RNAP holoenzymes containing either σ^A^ or σ^B^ can transcribe housekeeping genes in exponentially growing mycobacteria (56). Co-immunoprecipitated RNAs were isolated, strand-specific RNA libraries were prepared and sequenced by Illumina NextSeq.

To determine the enriched RNAs that interact with RNAP or σ^A^/σ^B^, we calculated the ratios of RNAP or σ^A^/σ^B^ reads in immunoprecipitation versus input samples (total RNA isolated from the lysate) for each annotated transcript (Figure 1F–I) or intergenic regions (Supplementary Figure S2) by DESeq2. The intergenic regions were used to reveal unannotated RNAs. First, we calculated the immunoprecipitation versus input ratios for each transcript or intergenic region. This ratio was then normalized to the rRNA immunoprecipitation versus input ratio that represents the non-specific signal (threshold line labeled in grey in Figure 1F–I and Supplementary Figure S2). Normalization to rRNAs was used to merge data from different replicates and to compare results of different bacterial species or growth phases. As an additional reference point, tmRNA is shown. This structured RNA, which shares similar expression levels and length with Ms1 RNA, does not bind to either RNAP or σ^A^/σ^B^ (57,58) and can be thus used as a negative control. Supplementary Table S1 shows counts of mapped reads for genes/intergenic regions and fold-change and FDR-corrected *P*-value plotted in Figure 1 and Supplementary Figure S2. Native RNA immunoprecipitation allowed us to determine the complete set of RNAs associated with RNAP or σ^A^/σ^B^, including transcripts with low levels of expression.

Ms1 RNA was strongly associated with RNAP in stationary phase (Figure 1G), but in the stationary phase σ^A^/σ^B^ RIP-seq dataset, Ms1 RNA enrichment was comparable to tmRNA that does not interact with RNAP or σ^A^/σ^B^ (Figure 1I). In the exponential phase σ^A^/σ^B^ RIP-seq dataset, Ms1 was enriched ∼4.5 fold. However, more than 6300 transcripts showed higher enrichment than Ms1 RNA (Figure 1H and Supplementary Table S1), confirming that Ms1 is not 6S RNA (33). Overall, the failure to detect a ncRNA with features of 6S RNAs by σ^A^/σ^B^ RIP-seq in *M. smegmatis* further substantiates the lack of a 6S homolog in this bacterium.

**Figure 2.**
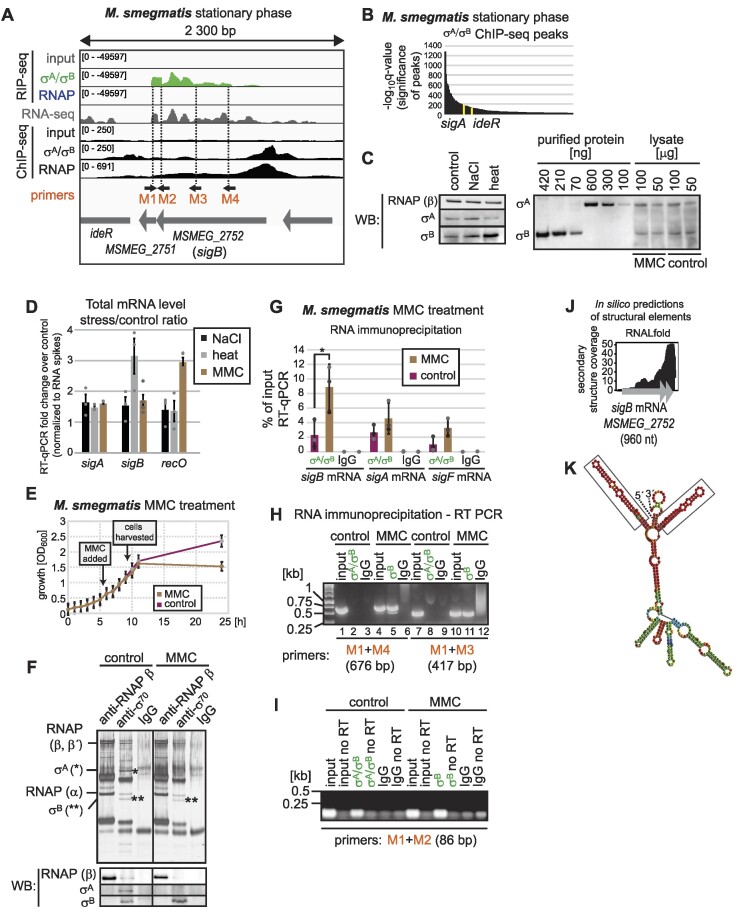
The interaction between σ^A^/σ^B^ protein and *sigB* mRNA is affected by MMC. (**A**) Stationary phase RIP-seq and ChIP-seq data from *M. smegmatis* for the *sigB* gene. RNA-seq data was published previously (34). ChIP-seq detects genomic DNA sequences where the transcribing or stalled RNAP-σ holoenzymes or RNAPs are enriched. The position of primers used in RT-PCR in **H** and **I** are shown. (**B**) σ^A^/σ^B^ peaks detected by ChIP-seq in stationary phase. (**C**) The level of σ^A^ and σ^B^ in total protein lysates after NaCl, heat and mitomycin C (MMC) stresses in *M. smegmatis* as detected by western blotting. The level of σ^A^ and σ^B^ protein changes after treating cells with NaCl and heat but not after MMC treatment. The amounts of σ^A^ and σ^B^ were detected with the anti-σ^70^ antibody, purified σ^A^ and σ^B^ were used as positive control. The anti-RNAP antibody was used to detect the level of RNAP. (**D**) mRNA levels of *sigA, sigB* and*recO* in NaCl, heat and MMC treated cells. The y-axis shows the mRNA level in stressed cells that was normalized to the control cells, which were set as 1. Spike-in RNAs were added at the beginning of the RNA isolation (see Materials and methods) and RT-qPCR was normalized to spike-in RNAs. *recO* (*MSMEG_4491*) mRNA was used as a positive control for MMC treatment (67). The data shows the average value from three independent replicates, error bars represent SEM. (**E**) Growth curves of *M. smegmatis* in the presence or absence of mitomycin C (MMC), the data shows average values from three independent replicates, error bars represent SEM. (**F**) In MMC-treated cells, no σ^A^ was pulled down with the anti-σ^70^ antibody. The pulled-down proteins were resolved on SDS-PAGE, silver-stained and their identities were confirmed by MALDI mass spectrometry. σ^A^ and σ^B^ were detected also by western blotting with the anti-σ^70^ antibody. (**G**) *sigB* transcript was enriched in RNA immunoprecipitations with anti-σ^70^ antibody after MMC treatment. The data shows the average values from three independent replicates, error bars represent SEM. The asterisk indicates a significant difference as detected by paired *t*-test (*P*-value 0.03). (**H, I**) The presence of long *sigB* RNAs in MMC-treated cells was confirmed by RT-PCR with different primer pairs (shown in A). (**J**) Stable secondary structures were detected at the 3′end of *sigB* RNA by RNALfold. The secondary structure coverage displays the numbers of detected short, stable secondary structures (y-axis) along the *sigB* mRNA sequence. (**K**) The secondary structure of a fragment of *sigB* mRNA predicted by RNAfold (Vienna RNAfold webserver). The parts of the secondary structure with the highest base pair probabilities are highlighted in red. The locally stable secondary structures detected by RNALfold are marked by rectangles. The presented fragment of *sigB* mRNA was selected based on RIP-seq enrichment and has the following genome coordinates: NC_008596.1: 2822589–2822224.

While we did not detect any 6S RNA in *M. smegmatis* (Figure 1H, I and Supplementary Figure S2C and D), we detected the binding of σ^A^/ σ^B^ to transcripts encoding three σ factors – *sigB*, *sigA* and *sigF* (Figure 1H and I). Figure 2A and Supplementary Figure S3A and C show a detailed view of the RIP-seq signal across these genomic loci. The *sigB* transcript (*MSMEG_2752* gene) was enriched in σ^A^/σ^B^ RIP-seq about 220-fold in exponential and 583-fold in stationary phase (Supplementary Table S1). RNA derived from the *MSMEG_2751* gene, which is the 3′ end flanking gene of *sigB*, was enriched in σ^A^/σ^B^ RIP-seq approximately 370-fold in exponential and 680-fold in stationary phase (Supplementary Table S1). *MSMEG_2751* encodes a hypothetical 50 amino acid protein (59), but this protein is neither annotated in the most recent *M. smegmatis* NCBI annotation (NC_008596.1) nor in *M. tuberculosis* and very probably represents the 3′ UTR of *sigB* mRNA. The interaction of σ^A^/σ^B^ with *sigA* (*MSMEG_2758*) and *sigF* (*MSMEG_1804*) transcripts was detected only in stationary phase (Figure 1I).

In addition, σ^A^/σ^B^ was bound to transcripts derived from the *MSMEG_1253* gene of unknown function and from the *ideR* gene (*MSMEG_2750*), which is positioned downstream of *MSMEG_2751* and probably represents readthrough transcripts of *sigB* mRNA that is highly associated with σ^A^/σ^B^ proteins. In the intergenic regions, sequences flanking the 5′ or 3′ ends of *sigB*, *sigA, sigF* and *MSMEG_1253* genes were enriched on σ^A^/σ^B^ in RIP-seq (Supplementary Figure S2C and D). Figure 2A and Supplementary Figures S3A, B and C provide a detailed view of the RIP-seq signal across these genomic loci.

To exclude the possibility that *sigB*, *sigA*, *sigF* and *MSMEG_1253* transcripts are nascent RNAs physically coupled to the transcribing σ^A^/σ^B^-RNAP holoenzymes, we performed chromatin immunoprecipitation and sequencing (ChIP-seq) with the same anti-σ^70^ and anti-RNAP β antibodies to determine the distribution of RNAP and RNAP-σ^A^/σ^B^ complexes in the genome. Then, we looked at the correlation between the RIP-seq and ChIP-seq data. If an RNA/gene was highly enriched in both datasets, it was likely a transcript emerging from transcribing RNAP. If RIP-seq > ChIP-seq, then the transcripts were mainly associated with the free σ^A^/σ^B^ or σ^A^/σ^B^-RNAP holoenzymes that were not bound to the chromosome.

In stationary ChIP-seq data, no significant σ^A^/σ^B^ peaks were detected at the *sigF* and *MSMEG_1253* genes (Supplementary Figure S3A and B and Supplementary Table S2). The promoters of *sigA* and *sigB* were occupied by σ^A^/σ^B^ (Supplementary Figure S3C and Figure 2A) but in RIP-seq, the enriched *sigA* and *sigB* transcripts also comprised the mRNA 3′ ends. Another point supporting the probable posttranscriptional association of σ^A^/σ^B^ with cognate mRNAs is the distribution of RNAP-bound ChIP-seq reads across these loci. This distribution is more clustered towards the 5′ end of the genes, displaying a pattern distinct from that observed in the RIP-seq σ^A^/σ^B^ track (Supplementary Figure S3A–C). In ChIP-seq, no σ^A^/σ^B^ was present at the 3′end of the *sigA* gene (Supplementary Figure S3C). The *sigA* transcript enriched in RIP-seq thus cannot represent the nascent RNA that is physically associated with the elongating RNAP core lacking the σ factor. ChIP-seq identified a small, but statistically significant peak in the region downstream of the *sigB* gene (Supplementary Table S2 and Figure 2A) which overlaps with the promoter of the *ideR* gene. This σ^A^/σ^B^ peak is not among the most significant peaks detected in stationary phase ChIP-seq (Figure 2B) and probably represents the association of σ^A^/σ^B^ with the promoter of the *ideR* gene. To conclude, σ^A^/σ^B^-associated transcripts detected in RIP-seq interact either with free σ^A^/σ^B^ proteins or with non-transcribing σ^A^/σ^B^-RNAP holoenzymes.

To provide a comprehensive and scalably detailed overview of the data, we built a web page with an integrated igv.js genome browser (60) to visualize *M. smegmatis* RIP-seq and ChIP-seq data. This information is available on the ‘msmegseq.elixir-czech.cz’ website.

### Interaction between σ^B^ protein and *sigB* RNA increases in DNA damage stress

In comparison to the exponential phase, an increase in the interaction between σ^A^/σ^B^ proteins and the *sigB* transcript during stationary phase was observed (∼580-fold versus ∼220-fold enrichment, Supplementary Table S1), along with other σ factor transcripts, *sigA* and *sigF* (Figure 1I). This suggests that σ^A^/σ^B^ increasingly bind to σ transcripts under stress conditions. We tested the interaction of σ^A^/σ^B^ proteins with σ factor transcripts in three different stress conditions—osmotic stress (0.5 M NaCl), heat stress (45°C) and DNA damage. To induce DNA damage, we treated *M. smegmatis* with mitomycin C (MMC), which causes inter strand crosslinks in DNA (61,62). DNA damage repair mechanisms are extensively studied in *M. tuberculosis*, because activated macrophages produce reactive oxygen species and nitric oxides that damage mycobacterial nucleic acids during infection (63).

To accurately measure the interaction between σ^A^/σ^B^ proteins and σ factor transcripts in various conditions, we first examined whether the expression of σ^A^/σ^B^ proteins and *sigB, sigA* and *sigF* transcripts was comparable. In NaCl-treated and heat-stressed cells, the amount of σ^B^ protein was partially increased, while the levels of both σ^B^ and σ^A^ proteins remained stable in MMC-treated cells (Figure 2C). Additionally, the level of *sigB* mRNA was also increased in heat-stressed cells (Figure 2D) as reported previously for heat-stressed *M. tuberculosis* (64–66). An increase in *recO* mRNA, which was used as a positive control for DNA damage (67), was also observed in MMC treated cells (Figure 2D). As observed in previous studies (68), a notable decline in the growth of bacterial cells subjected to osmotic and heat stress was recorded. The stresses were induced in the mid-exponential phase at OD_600_∼0.5, and after 4 h of osmotic and heat stress, cells reached OD_600_∼0.7 and ∼0.72, respectively, compared to OD_600_∼1.4 in control cells. During the initial hours of MMC treatment, the impact of MMC on mycobacterial growth was only minor (Figure 2E). As the interaction between σ^A^/σ^B^ proteins and the *sigB, sigA* and *sigF* transcripts depends on the growth phase, we decided to first proceed with MMC-treated cells.

After MMC treatment, the anti-σ^70^ antibody immunoprecipitated only σ^B^. Interestingly, σ^A^ was not detected in silver-stained gels or by western blotting (Figure 2F), although σ^A^ protein was still present in MMC-treated cells (Figure 2C). This could be due to the association of the σ^A^ containing RNAP holoenzyme with the PafBC heterodimer (67,69). Interaction with PafBC might block recognition of the σ^A^ epitope by the anti-σ^70^ antibody, which may explain why σ^A^ protein was not immunoprecipitated by the anti-σ^70^ antibody under these conditions (Figure 2F).

We measured the amounts of *sigB*, *sigA* and *sigF* transcripts that were bound to σ^B^ protein in MMC-treated cells by RT-qPCR. The association of σ^B^ protein with the *sigB* transcript was significantly increased in MMC-treated cells (Figure 2G). We also observed a ∼50% increase of the total *sigB* transcript level in MMC-treated cells compared to non-treated controls (Figure 2D), however the total amount of σ^B^ protein was unchanged (Figure 2C). In MMC-treated cells, translation of *sigB* mRNA could be negatively affected by its association with σ^B^ protein, therefore, we tested if σ^B^ binds to the entire *sigB* mRNA or to a fragment thereof.

Using semi-quantitative RT-PCR, we confirmed that σ^B^ associates with *sigB* mRNA (Figure 2H, lanes 5 and 11) in MMC-treated cells. We did not detect longer parts of *sigB* mRNA bound to σ^B^ (or σ^A^) in non-treated cells (Figure 2H, lanes 2 and 8), only the 3′end of *sigB* was bound (Figure 2I). At the 3′end of the *sigB* RNA sequence, we also detected locally stable secondary structures using RNALfold (49) (Figure 2J). The predicted secondary structure of the entire *sigB* fragment is shown in Figure 2K and the main structures which were identified by RNALfold have been highlighted in boxes. Thus, the 3′end of the *sigB* transcript appears to represent a putative regulatory sequence that is responsible for the σ^B^ (and/or possibly σ^A^) protein-*sigB* RNA interaction in untreated cells. Therefore, we conclude that the σ^B^ protein binds its own *sigB* transcript in cells stressed by DNA damage.

To identify additional proteins that might be a part of σ^A^/σ^B^–*sigB* mRNA complex in non-treated cells, we performed a mass spectrometry analysis of anti-σ^70^ antibody immunoprecipitates from stationary phase *M. smegmatis* cells. Major proteins present in these anti-σ^70^ antibody immunoprecipitates and absent from IgG negative controls are shown in Supplementary Table S3. These proteins included RNAP subunits (α, β, β′), σ factors (σ^A^, σ^B^, σ^F^), RbpA protein, Rho factor and proteins from small or large ribosomal subunits (most probably due to the coupling of transcription and translation). Excluding RNAP subunits and σ^B^, which are clearly visible upon silver-staining, none of the additional proteins were detected in silver-stained gels showing the co-immunoprecipitated proteins with the anti-σ^70^ antibody in MMC-treated cells (Figure 2F), in which the interaction between the σ^B^ and *sigB* transcript was elevated (Figure 2G). However, we cannot exclude the possibility that any of these proteins participates in the σ^A^/σ^B^ protein and *sigB* transcript interaction.

### Interaction between σ^A^/σ^B^ protein and *sigB* RNA does not increase in osmotic and heat stress

**Figure 3.**
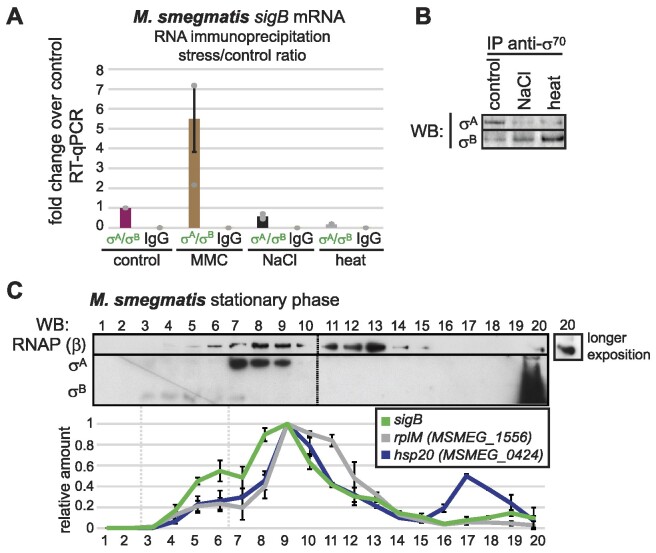
The interaction between *sigB* mRNA and σ^A^ and σ^B^ proteins is not enhanced in NaCl and heat stress and *sigB* mRNA co-sediments with σ^B^ protein. (**A**) *sigB* transcript was enriched in RNA immunoprecipitations with anti-σ^70^ antibody after MMC treatment, while in NaCl and heat stress, *sigB* mRNA interaction with σ^A^ and σ^B^ proteins did not increase. The y-axis shows the % of input in control and stressed cells that was normalized to the control cells, set as 1. The data shows the average values from three independent replicates, error bars represent SEM. (**B**) Western blotting of the immunoprecipitated σ^A^ and σ^B^ proteins from control cells and cells subjected to NaCl and heat stress, detected by the anti-σ^70^ antibody. (**C**) Total protein lysates from *M. smegmatis* stationary phase cells were separated by glycerol gradient ultracentrifugation. The amounts of RNAP and σ^A^ and σ^B^ in individual fractions were detected by western blotting using anti-RNAP and anti-σ^70^ antibodies, respectively. The amount of *sigB* mRNA in individual fractions was quantified using RT-qPCR. *rplM* (*MSMEG_1556*) and *hsp20* (*MSMEG_0424*) mRNAs were used as negative controls. Note that compared to negative controls, *sigB* mRNA was enriched in fractions 5 and 6, co-sedimenting with the free σ^B^ protein.

In contrast to DNA damage stress, osmotic stress (NaCl) and heat stress did not increase the enrichment of *sigB* transcript on σ^A^/σ^B^ proteins (Figure 3A). The anti-σ^70^ antibody immunoprecipitated more σ^B^ protein than σ^A^ in NaCl- and heat-stressed cells compared to control cells (Figure 3B). In MMC treated cells, the anti-σ^70^ antibody also immunoprecipitated more σ^B^ protein than what was found in control cells (Figure 2F, see western blot at the bottom). However, the amount of the enriched *sigB* transcript was increased in MMC treated cells and decreased in NaCl- and heat-stressed cells (Figures 2G and 3A). Therefore, the amount of the pulled-down *sigB* transcript does not depend solely on the amount of the pulled-down σ^B^ protein. In NaCl- and heat-stressed cells, while σ^B^ was efficiently translated, leading to an increase in σ^B^ levels (Figure 2C), its interaction with the *sigB* transcript was reduced (Figure 3A) in contrast to the scenario observed in the MMC-treated cells.

Recently, a supramolecular, transcriptionally inactive complex composed of eight *M. tuberculosis* σ^B^-RNAP holoenzyme units was described in *vitro* (70). Therefore, we speculated that the *sigB* transcript might associate only with a specific fraction of σ^B^ protein-containing complexes. To distinguish different σ^B^ complexes, we fractionated lysates from stationary phase cells by ultracentrifugation in glycerol gradients in a manner similar to what we have done previously to detect Ms1-RNAP (33). Simultaneously, we isolated RNA from each fraction of the gradient and measured the level of *sigB* and two control transcripts that were not enriched in σ^A^/σ^B^ RIP-seq (*rplM* mRNA encoding a ribosomal protein gene and *MSMEG_0424* encoding Hsp20/alpha crystallin family protein heat shock protein). The majority of the σ^B^ protein sedimented at the bottom of the gradient (fraction 20, Figure 3C) where RNAP was also detected. However, *sigB* transcripts remained nearly undetectable in fraction 20 of the gradient (Figure 3C), suggesting that *sigB* transcripts are not included in this putative macromolecular complex. Unlike *rplM* and *MSMEG_0424* mRNAs, the *sigB* transcript showed partial co-sedimentation with the σ^B^ protein specifically in fractions 5–7 (Figure 3C). Hence, the *sigB* transcript may act as a ‘sensor’ that perceives the levels of free σ^B^ or the σ^B^-containing holoenzyme, but additional data will be needed to support this hypothesis.

Therefore, the bacterial transcription machinery interacts not only with abundant, structured regulatory RNAs (such as 6S or Ms1 RNA), but also with specific mRNA transcripts. These interactions are highly selective since only several transcripts were enriched in RIP-seq (Figure 1).

### A fragment of *recO* transcript binds to RNAP

**Figure 4.**
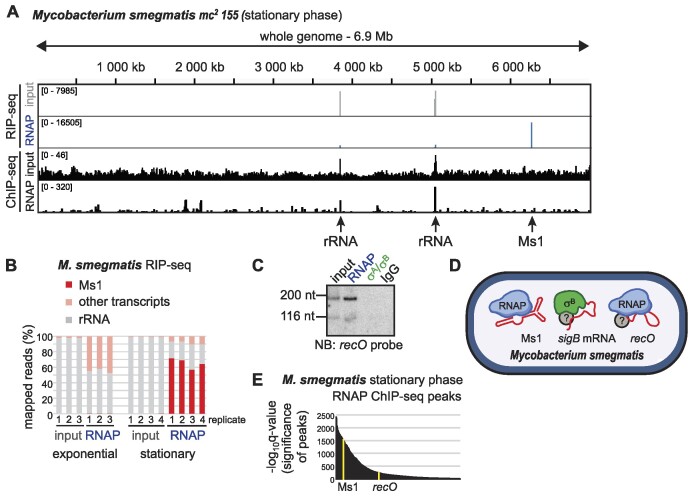
Ms1 is the major RNAP-interacting RNA in *M. smegmatis*. (**A**) Example of RIP-seq and ChIP-seq data from *Mycobacterium smegmatis* stationary phase, whole genome view. In the RNAP RIP-seq sample, most of the reads mapped to Ms1. (**B**) Quantification of RIP-seq data. In stationary phase, >60% of reads mapped to Ms1, while in exponential phase, RNAP binds mainly to rRNAs or other mRNAs that either represent actively transcribed nascent RNAs or background of non-specifically bound RNAs. (**C**) *recO* RNA fragments specifically bind RNAP. *recO* RNA was detected by northern blotting, for the position of the *recO* probe, see Supplementary Figure S3D. (**D**) Summary of RNAs that bind to the transcription machinery in *M. smegmatis* that we detected by RIP-seq. Question marks indicate that the exact composition of individual RNA-protein complexes is not known. (**E**) RNAP peaks detected by ChIP-seq in stationary phase. RNAP signal was detected on *recO* gene, but the exact position of RIP-seq peaks along the gene differs from the ChIP-seq peaks position (Supplementary Figure S3D), indicating that free and not co-transcriptionally associated *recO* RNAs are bound to RNAP.

In the RIP-seq input sample (total RNA isolated from the lysate) from stationary *M. smegmatis* cells, most sequenced reads mapped to the two rRNA operons as expected (Figure 4A, RIP-seq input, an overview of mapped reads on the whole genome is shown). In the RIP-seq RNAP sample (RNAs that associate with RNAP), however, >60% of reads mapped to Ms1 (Figure 4A, B). Thus, in stationary phase, RNAP binds mainly to Ms1, while in exponential phase, RNAP binds mainly to rRNAs or mRNAs (Figure 4B) that represent either nascent transcripts coupled to the transcribing RNAP complexes or a non-specific background. Therefore, Ms1 RNA has the potential to sequester RNAP but only in stationary phase.

In addition to Ms1, a ∼200 nt fragment of the *recO* transcript *(MSMEG_4491)* binds to *M. smegmatis* RNAP in stationary phase (Figure 1G and Supplementary Figure S3D) but not to σ^A^/σ^B^ proteins (Figure 1I). The *recO* mRNA is poorly expressed (see the ‘Mean normalized expression in input’ in Figure 1G) and the amount of *recO* fragment appears too low to bind a significant amount of RNAP. The *recO*-RNAP interaction was confirmed by northern blotting (Figure 4C). The predicted secondary structure of the *recO* fragment is shown in Supplementary Figure S3F and does not resemble Ms1 or 6S RNA. While we detected the long *sigB* mRNA to bind to the σ^B^ protein after MMC treatment (Figure 2H), only a short *recO* mRNA fragment bound RNAP both in control and MMC-treated cells (Supplementary Figure S3E). This suggests that the bacterial transcription machinery interacts either with mRNAs (such as *sigB* mRNA) or with specific short mRNA fragments (*recO*) (Figure 4D) and these interactions are differentially regulated during stress conditions, such as MMC.

Based on the ChIP-seq data, RNAP is located at many gene promoters in stationary phase (Figure 4A and E). In the case of *recO*, the ChIP-seq peak is positioned more towards the promoter and the transcription start site of the gene while the RIP-seq peak is shifted to the coding region (Supplementary Figure S3D). This implies that the *recO* signal in RNAP RIP-seq does not represent the nascent transcript coupled to the transcribing RNAP suggesting that *recO* binds to the free RNAP.

### MTS2823 sRNA binds RNAP in *Mycobacterium tuberculosis*

MTS2823 is a highly abundant sRNA in stationary phase in *M. tuberculosis* (35); an even higher accumulation of MTS2823 sRNA was observed in mice during chronic infection of tuberculosis (35). Based on the nucleotide sequence, MTS2823 is an Ms1 homolog (33,36) but it has never been shown to associate with the RNAP core in *M. tuberculosis*. Therefore, we performed RIP-seq with anti-σ^70^ and anti-RNAP antibodies in the *M. tuberculosis H37Rv* laboratory strain.

The anti-RNAP antibody pulled down three main subunits of RNAP core (β, β′, α) (Supplementary Figure S4A) and the results from western blotting did not provide evidence for the presence of σ^A^ (Supplementary Figure S4B). The anti-σ^70^ antibody pulled down σ^A^, but also RNAP β, β′, indicating that the primary σ factor-containing holoenzyme was immunoprecipitated (Supplementary Figure S4A and B). Although we did not detect the σ^B^ protein in anti-σ^70^ antibody immunoprecipitation (Supplementary Figure S4A), we cannot exclude that σ^B^ is present albeit in low levels because the σ^B^ epitope, which is recognized by anti-σ^70^ antibody, is conserved in both, *M. smegmatis* and *M. tuberculosis* (Supplementary Figure S1).

**Figure 5.**
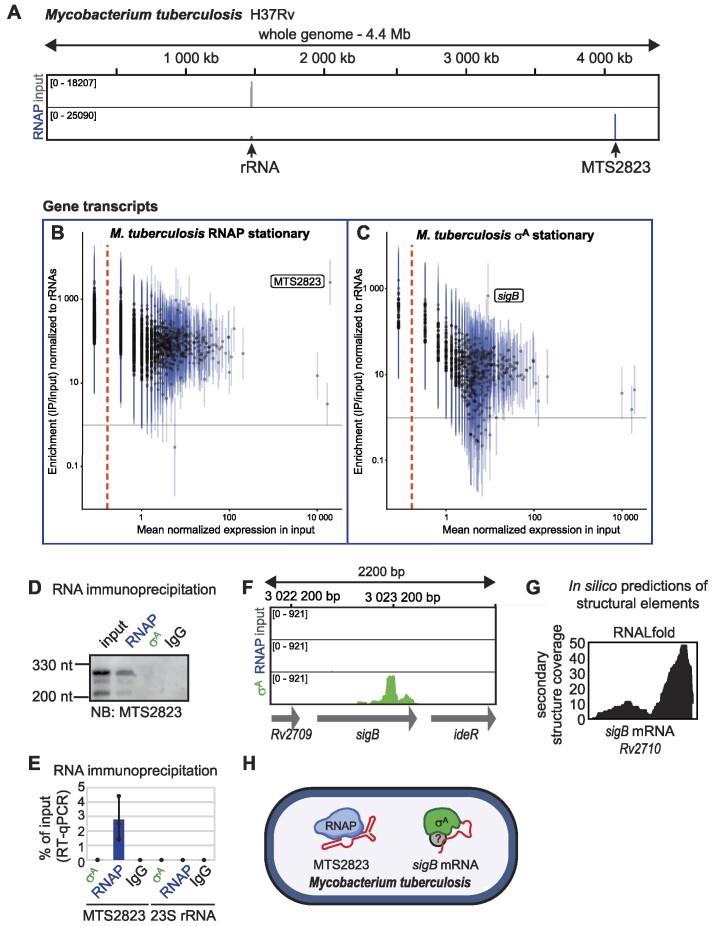
RIP-seq in *Mycobacterium tuberculosis*. (**A**) RIP-seq data for *Mycobacterium tuberculosis* H37Rv, whole genome view. In the RNAP RIP-seq sample, most of the reads mapped to MTS2823 RNA. (**B**, **C**) Quantification of RIP-seq data from *M. tuberculosis* in stationary phase for RNAP (**B**) and σ^A^ (**C**) for each annotated gene. For further details, see legend to Figure 1. For intergenic regions, please see Supplementary Figure S4. (**D, E**) The binding of MTS2823 RNA to RNAP was confirmed by northern blotting and RT-qPCR. (**F**) High amounts of the *sigB* transcript were detected in the σ^A^ RIP-seq sample. (**G**) Stable secondary structures were detected at the 3′end of *sigB* mRNA by RNALfold. (**H**) Summary of RNAs that bind to the transcription machinery in *M. tuberculosis* as detected by RIP-seq. Question marks indicate that the exact composition of individual RNA-protein complexes is not known. Although σ^B^ has not been detected in the immunoprecipitations with anti-σ^70^ antibody (see Supplementary Figure S4A), we cannot rule out the possibility that it is present among the pulled-down proteins in low amount.

As in *M. smegmatis* Ms1, the majority of sequenced reads mapped to the MTS2823 genome locus in the *M. tuberculosis* RNAP RIP-seq sample (Figure 5A). No other transcript was enriched in RNAP RIP-seq (Figure 5B), although we detected a signal of RNAP at a ∼260 nt transcript derived from the *Rv1534*-*Rv1535* intergenic region (Supplementary Figure S4C, D). *Rv1535* encodes a hypothetical protein and overlaps with the long 5′UTR of the *ileS* gene encoding isoleucyl-tRNA synthase. Although a strong RNAP signal was visible in RIP-seq at the ∼260 nt transcript (Supplementary Figure S4C), this transcript is highly expressed (among top 10 most abundant RNAs, Supplementary Table S4) and therefore is not significantly enriched on RNAP compared to other less expressed gene or intergenic transcripts (see fold change over input in Supplementary Figure S4D and Supplementary Table S4). We did not detect any association of the *recO* homolog (*Rv2362c*) with RNAP. We confirmed the association of MTS2823 with RNAP by northern blotting and RT-qPCR (Figure 5D and E), but MTS2823 did not bind σ^A^, and by inference σ^A^-RNAP (Figure 5D and E). These findings show that Ms1/MTS2823 interaction with the RNAP core is conserved in pathogenic mycobacteria.

We observed an enrichment of the *sigB* transcript (*Rv2710*) in σ^A^ RIP-seq (Figure 5F). Therefore, not only the Ms1/MTS2823-RNAP interaction but also the σ^A^/σ^B^–*sigB* RNA interaction is conserved in mycobacteria. In addition, secondary structures are present at the 3′end of *sigB* mRNA in *M. tuberculosis* (Figure 5G) as well as in *M. smegmatis* (Figure 2J and K).

Thus, in addition to evolutionarily conserved RNAs binding to the transcriptional machinery, mycobacteria have evolved species-specific RNA–RNAP/σ^A^/σ^B^ interactions (Figures 4D and 5H).

### scr3559 and scr0792 RNAs interact with the transcription machinery in *Streptomyces coelicolor*

Both mycobacteria and streptomyces belong to the phylum actinobacteria and are evolutionarily related. Nevertheless, mycobacteria are rod-shaped and unicellular, while streptomyces have a complex life cycle that starts with spore germination, then proceeds to a filamentous and highly branched vegetative (primary) mycelium, followed by formation of a secondary mycelium and eventually spores. Recently, there were contradictory results describing the presence of 6S RNA or an Ms1 homolog in *Streptomyces coelicolor* (30,31,37).

**Figure 6.**
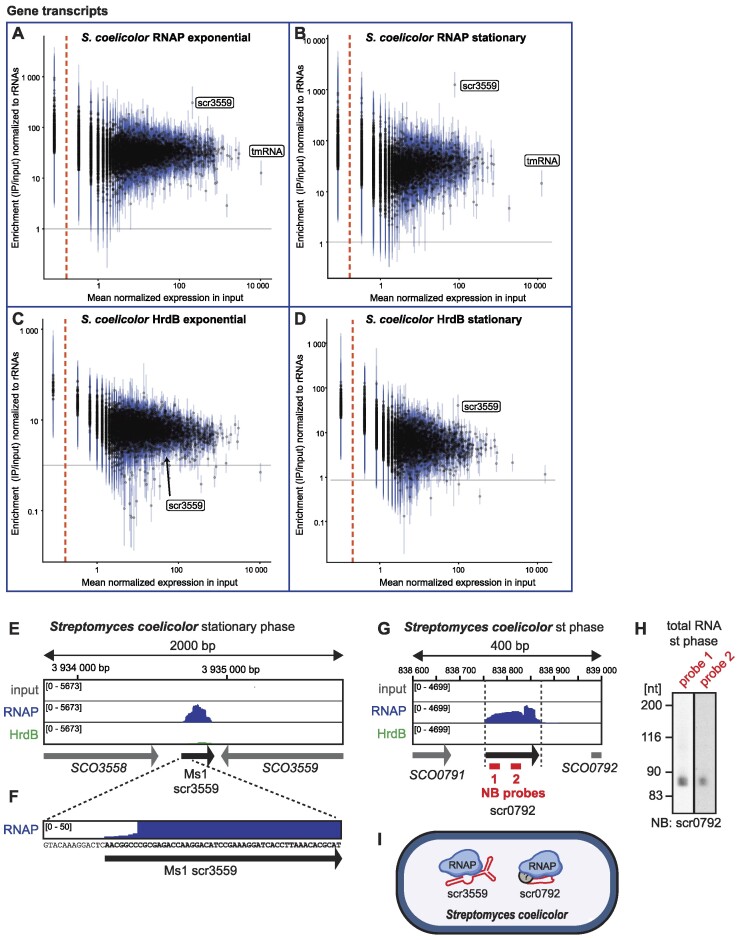
RIP-seq in *Streptomyces coelicolor*. Quantification of RIP-seq data for RNAP (**A, B**) and HrdB (**C, D**) for each annotated gene in *S. coelicolor* in exponential and stationary phase. For intergenic regions, please see Supplementary Figure S5. For further details, see legend to Figure 1. (**E**) RIP-seq using *S. coelicolor* cells in stationary phase revealed the scr3559 transcript (Ms1 homolog) to strongly enrich with RNAP, but there are also some reads mapping to scr3559 RNA in HrdB RIP-seq samples (in green). (**F**) The first nucleotide of scr3559 RNA that associates with RNAP in stationary phase of growth corresponds to the first nucleotide of the scr3559 transcript as defined by 5′RACE using total RNA and published previously (37). (**G**) scr0792, a novel transcript from the intergenic region between the genes *SCO0791* and *SCO0792* binds to RNAP in stationary phase of growth in *S. coelicolor*. The position of the two probes that were used for northern blotting are shown. (**H**) The two independent probes complementary to scr0792 RNA detected a ∼85 nt long RNA in stationary *S. coelicolor* cells. (**I**) Summary of RNAs that bind to the transcription machinery in *S. coelicolor* as detected by RIP-seq. Question marks indicate that the exact composition of individual RNA-protein complexes is not known.

Therefore, we also focused on RNAs associated with the transcriptional machinery in *S. coelicolor* A3(2) *hrdB-*HA, a strain in which the primary σ factor (HrdB) is endogenously tagged with hemagglutinin (HA) (38). We had previously shown that the anti-HA antibody immunoprecipitated the primary σ factor holoenzyme (HrdB–HA in complex with RNAP) (37). Here, we performed RNAP and HrdB-HA RIP-seq from exponential and stationary phases of growth (42 h and 66 h after germination, respectively, Figure 6; Supplementary Table S5, for details, see the Supplementary material).

We confirmed that scr3559 sRNA is an Ms1 homolog in *S. coelicolor* (37) (Figure 6A and B) and RIP-seq also verified the first nucleotide of scr3559 (Figure 6E and F) which we had determined by 5′ RACE previously (37). In exponential phase, scr3559 strongly interacted with RNAP (∼310-fold enrichment) and did not interact with HrdB (∼2-fold enrichment, Figure 6A and C, Supplementary Table S5). In stationary phase, scr3559 was highly enriched on RNAP (∼1250-fold) and we also observed a weaker accumulation with HrdB (Figure 6B and D). The enrichment on HrdB was only ∼45-fold (Supplementary Table S5), indicating that although a minor fraction of scr3559 is able to associate with RNAP-HrdB, the majority of scr3559 binds to RNAP without HrdB.

We identified a novel RNA which is transcribed from the *SCO0791*_*SCO0792* intergenic locus and binds to RNAP in stationary phase (712-fold enrichment, Supplementary Table S5, Supplementary Figure S5B and Figure 6G). We named this sRNA scr0792 according to the established nomenclature (71). We validated scr0792 expression by northern blotting using two independent probes and estimated that scr0792 has a length of ∼85 nt (Figure 6H). scr0792 is shorter than any known 6S or Ms1 RNA; one of the shortest known 6S RNAs is the ∼160 nt long 6S RNA from the hyperthermophilic bacterium *Aquifex aeolicus* (72). The secondary structure prediction of scr0792 is shown in Supplementary Figure S5E.

In conclusion, *S. coelicolor* has two RNAP-associated RNAs, an Ms1 homolog, scr3559, and the short scr0792 sRNA (Figure 6I). In the tested growth conditions, we did not detect any RNA strongly associated with HrdB (Figure 6C and D, Supplementary Figure S5C and D).

### CoRP RNA binds both RNAP core and RNAP holoenzyme in *Corynebacterium glutamicum*

Corynebacteria are evolutionarily relatively close to mycobacteria (both are in the same order—*Corynebacteriales*), but neither Ms1 nor 6S RNA has been found in *Corynebacterium glutamicum* with searches based on gene synteny or RNA structural similarity (37). Therefore, we tested whether RIP-seq can reveal any Ms1/6S RNA homolog in *C. glutamicum*. The anti-σ^70^ antibody efficiently immunoprecipitated the primary σ factor σ^A^ and σ^A^-RNAP holoenzyme (Supplementary Figure S6A, the identity of protein bands in Coomassie stained SDS-PAGE was confirmed by MALDI mass spectrometry). The anti-σ^70^ antibody also pulled down σ^B^ (Supplementary Figure S6A and B). The epitope recognized by the anti-σ^70^ antibody is partially present in the σ^B^ protein (Supplementary Figure S6C). In addition, we performed LC-MS/MS analyses of the complete anti-σ^70^ antibody immunoprecipitate (Supplementary Table S6) and identified both σ^A^ and σ^B^.

The anti-RNAP antibody immunoprecipitated RNAP core subunits β, β′and α (Supplementary Table S7 and Supplementary Figure S6A). We detected σ^A^ in the complete anti-RNAP antibody immunoprecipitate by LC-MS/MS (Supplementary Table S7), but we were unable to prove the presence of σ^A^ in SDS-PAGE or by western blotting (Supplementary Figure S6A and B). This finding indicates that only limited amounts of σ^A^-RNAP or σ^B^-RNAP holoenzymes are pulled down by the anti-RNAP antibody.

**Figure 7.**
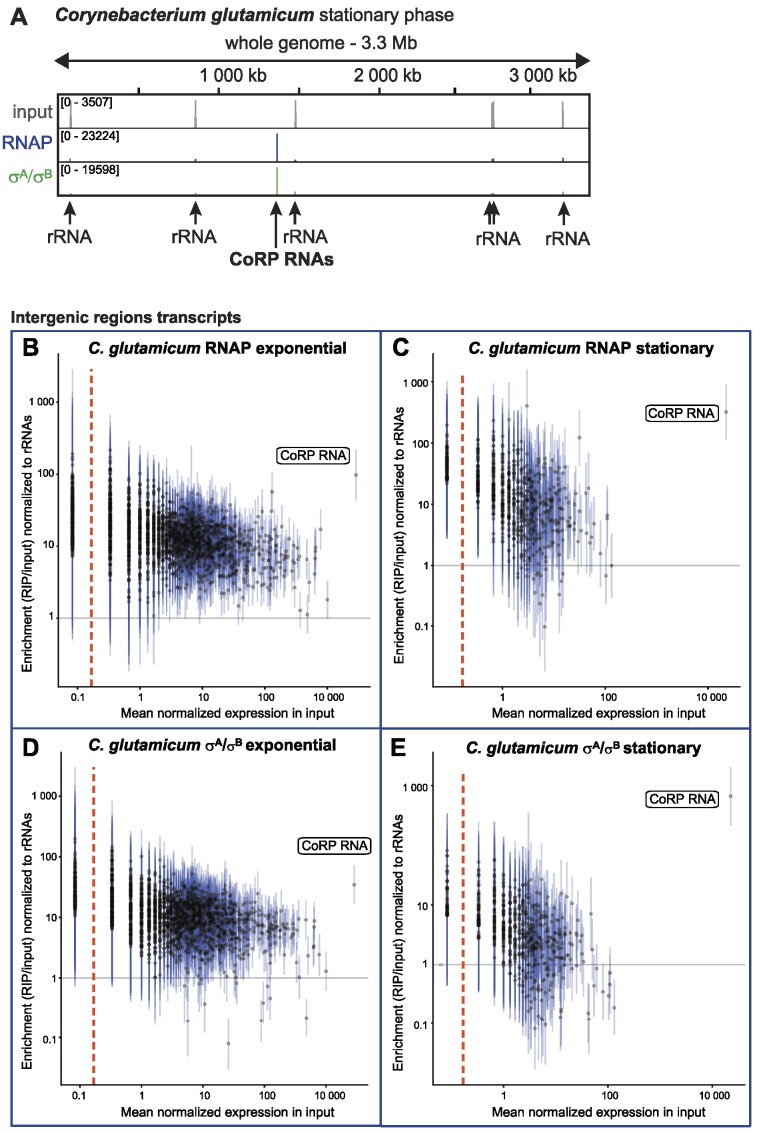
CoRP RNA associates with the transcription machinery in *C. glutamicum*. (**A**) RIP-seq using *C. glutamicum* cells in stationary phase. In stationary phase σ^A^ and RNAP RIP-seq samples, most of the reads mapped to the 1366239–1366719 intergenic region. The new RNA bound to σ^A^ and RNAP was named CoRP RNA. (**B**–**E**) Quantification of RIP-seq data for RNAP (**B, C**) and σ^A^/σ^B^ (**D, E**) for each intergenic region in the genome of in *C. glutamicum* in exponential and stationary phase. For further details, see legend to Figure 1. For annotated genes, please see Supplementary Figure S7.

**Figure 8.**
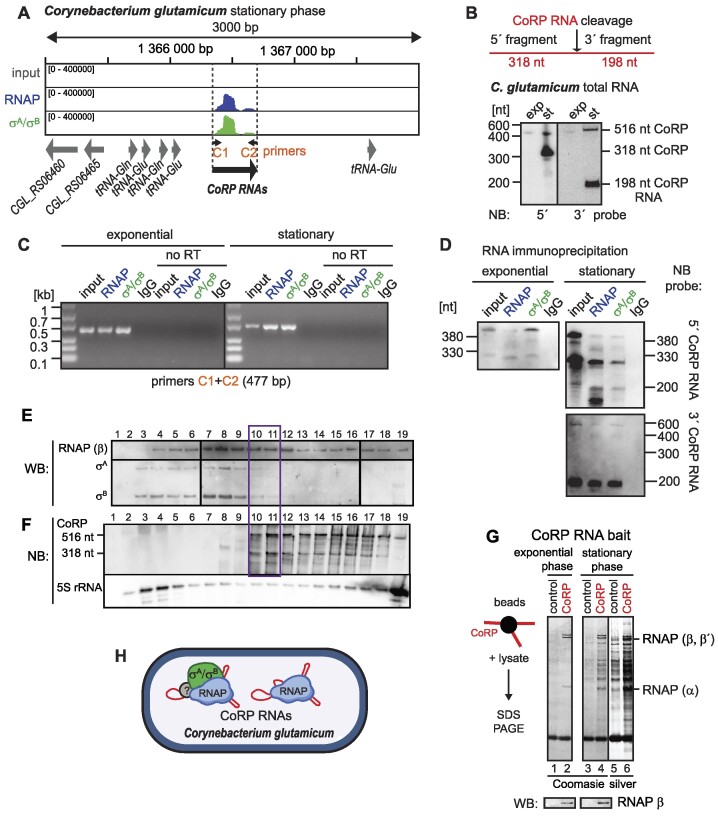
CoRP RNAs associate with RNAP holoenzyme and core in exponential and stationary phase. (**A**) Detailed view of the mapped reads at the CoRP RNA locus. 5′ and 3′flanking genes encode tRNAs. (**B**) CoRP RNA expression in *C. glutamicum* cells in exponential and stationary phase of growth. Based on 5′ and 3′RACE, the full-length CoRP RNA is cleaved into two fragments (5′ fragment 318 nt and 3′ fragment 198 nt). Two northern blot probes were used to detect the 5′ and 3′ fragments and the sizes of both fragments correspond to the cleavage site detected by RACE. (**C**) In both exponential and stationary phases, the full-length CoRP RNA interacts with σ^A^/σ^B^ and RNAP. The positions of primers used for RT-PCR are indicated in (**A**). (**D**) The association of the full-length CoRP RNA with σ^A^/σ^B^ and RNAP was also confirmed by northern blotting, although the majority of CoRP RNA bound to σ^A^/σ^B^ and RNAP is fragmented. Both 5′ and 3′ fragments interact with σ^A^/σ^B^ and RNAP in stationary phase. (**E**) Total protein lysates from *C. glutamicum* stationary phase cells were separated by glycerol gradient ultracentrifugation. The RNAP and σ^A^ and σ^B^ profiles across individual fractions were detected by western blotting using anti-RNAP and anti-σ^70^ antibodies, respectively. (**F**) The CoRP RNA was detected in individual fractions by northern blotting. The majority of the RNAP-σ^A^ holoenzyme sedimented in fractions 7–9, while fractions 10–11 (marked by a violet rectangle) contained both the RNAP-σ^A^ holoenzyme and CoRP RNA. No CoRP RNA was detected in the upper fractions of the gradient, where free proteins (including free σ^A^ and σ^B^) sediment. Most of the CoRP RNA was detected in fractions 12–18, accompanied by RNAP without σ^A^ or σ^B^, respectively. (**G**) The protein pull-down was performed using the full-length 516 nt CoRP RNA. *In vitro* transcribed CoRP RNA was coupled to streptavidin coated beads via a biotinylated antisense oligonucleotide and incubated with the lysates from exponential and stationary phase cells. The pulled-down proteins were resolved on SDS-PAGE and detected by Coomassie and silver staining. The identity of RNAP subunits was confirmed by western blotting using the anti-RNAP antibody. (**H**) CoRP RNAs bind both RNAP core and RNAP–σ^A^/σ^B^ holoenzyme in *C. glutamicum*. Question marks indicate that the exact composition of individual RNA-protein complexes is not known.

We sequenced the co-immunoprecipitated RNAs both from exponential and stationary phase and mapped the reads to the *C. glutamicum* genome (Figure 7A). For input RNA, reads mapped mainly to rRNA operons as expected. In σ^A^/σ^B^ and RNAP RIP-seq, the majority of reads mapped to an intergenic region with no annotated transcript, NC_003450.3:1366239–1366719 (Figures 7A and 8A). We named this RNA CoRP RNA (*Corynebacterium*RNAP binding RNA). CoRP RNA was the only abundant transcript significantly enriched on RNAP and σ^A^/σ^B^ both in exponential and stationary phase (Figure 7 and Supplementary Figure S7; Supplementary Table S8—for details, see the Supplementary material).

We mapped CoRP RNA 5′and 3′ ends by RACE. CoRP RNA is 516 nt long (sequence in Supplementary Figure S8B) and in stationary phase it is cleaved after the guanine at position 318. In exponential phase, only the 516 nt CoRP RNA is weakly detectable (Figure 8B). The full-length CoRP RNA associates with RNAP and σ^A^/σ^B^ both in exponential and stationary phase (Figure 8C). The two CoRP cleavage products (318 nt and 198 nt fragments) can also associate with RNAP and σ^A^/σ^B^ (Figure 8D).

To test whether CoRP RNA can bind to RNAP without σ^A^/σ^B^, we fractionated protein complexes from stationary phase *C. glutamicum* cells using glycerol gradient ultracentrifugation (Figure 8E). RNAP-σ^A^ and RNAP-σ^B^ holoenzymes were detected in the same fractions as CoRP RNA (fractions 10 and 11, labelled in violet rectangle, Figure 8E and F), but based on glycerol gradient sedimentation, most of the RNAP-σ^A^ and RNAP-σ^B^ holoenzymes were not bound to CoRP RNA (fractions 7–9). This is in contrast to *E. coli*, where 6S RNA binds the majority of RNAP-σ^70^ holoenzyme in stationary phase (8,9). Similarly to *M. smegmatis*, *C. glutamicum* RNAP was also found in other fractions of the gradient which were devoid of σ^A^ and σ^B^ proteins (fractions 12–19). The presence of CoRP RNA in these fractions indicates that CoRP RNA can also interact with the RNAP core lacking σ^A^/σ^B^ proteins.

As we identified many proteins in anti-σ^70^ and anti-RNAP antibody immunoprecipitates (Supplementary Tables S6 and S7), we decided to perform a reciprocal experiment to assess whether additional proteins are necessary for the RNAP- or σ^A^/σ^B^-CoRP interaction. Using *in vitro* transcribed 516 nt CoRP RNA coupled to beads via biotinylated antisense oligonucleotide, we pulled down RNAP subunits from *C. glutamicum* exponential and stationary phase lysates (Figure 8G). The identity of RNAP subunits was confirmed by western blotting (Figure 8G). The CoRP RNA pull down in stationary phase appeared to contain additional proteins (Figure 8G, lane 4). However, the same protein bands were detected in the negative control (beads with biotinylated oligonucleotide but without bait RNA as described previously (53)) when the gel was silver-stained (Figure 8G, lane 5). We therefore assume that these proteins bound non-specifically to the beads, suggesting a direct interaction only between CoRP RNA and the RNAP core. We did not detect σ^A^ and σ^B^ proteins; either these proteins were below the detection limit or specific conditions are required for their stable interaction(s) with CoRP RNA. This experiment combined with the ultracentrifugation in glycerol gradient results thus reveal that the majority of CoRP RNA is bound to RNAP core and CoRP RNA–σ^A^/σ^B^–RNAP complexes are either unstable or present in small quantities.

### CoRP RNA homologs in the *Corynebacterium* genus

CoRP RNAs resemble both 6S RNA (binds the primary σ-RNAP holoenzyme) and Ms1 (binds RNAP core) but the full-length CoRP RNA is longer than any known 6S or Ms1 (∼500 nt versus ∼200 nt or ∼300 nt, respectively). Supplementary Figure S8A shows the predicted secondary structure of the 516 nt CoRP RNA. With BLAST (73) and rboAnalyzer (74) we found close homologs of CoRP RNA only within the genus *Corynebacterium* (Supplementary Table S9). Based on the rboAnalyzer results we observed conserved synteny for CoRP homologs conforming to the order: tRNA Gln – tRNA Glu – CoRP – tRNA Glu (Supplementary Figure S9A).

To verify the presence of the putative CoRP transcript in *Corynebacteriales*, we explored publicly available datasets for *C. glutamicum* (75–78), *C. pseudotuberculosis* (79,80) and *C. diphtheriae* (81). We found the transcript in several *C. glutamicum* datasets (Supplementary Figure S9B), in *C. pseudotuberculosis* (Supplementary Figure S9C) and *C. diphtheriae* (Supplementary Figure S9D). In *C. diphtheriae*, the putative CoRP RNA homolog partially overlaps with hypothetical proteins DIP1120 (94 amino acids) and DIPRS24085 (54 amino acids) in different genome annotations. Therefore, we cannot exclude that the transcript is partially translated. Based on the previously published data from *C. diphtheriae* (82), expression of DIP1120 mRNA is dramatically decreased in Viable but Nonculturable (VBNC) cells that were stressed for several weeks at 4°C compared to growing cells. CoRP/DIP1120 RNA was then significantly upregulated when VBNC cells were resuscitated (82), indicating its importance for active growth.

Taken together, we have provided evidence that CoRP RNAs associate with both, the primary σ^A^ holoenzyme and RNAP core (Figure 8H), respectively, and are present in *C. glutamicum* not only in stationary phase but also during the exponential growth (Figure 8B–D).

### 6S-1 and 6S-2 RNAs associate with σ^A^–RNAP complex during exponential growth in *Bacillus subtilis*

Next, we decided to perform RIP-seq in the model organism *Bacillus subtilis* that has 6S-1 and 6S-2 RNA genes (reviewed in (7,83,84)) to explore whether 6S-1 or 6S-2 or any other RNA binds to the primary σ^A^ holoenzyme also in exponential phase. *B. subtilis* has two 6S RNAs, 6S-1 (*bsrA*) and 6S-2 (*bsrB*) with different expression profiles: 6S-1 is expressed mainly in stationary phase while 6S-2 expression peaks at the mid-exponential phase (22,28,85).

**Figure 9.**
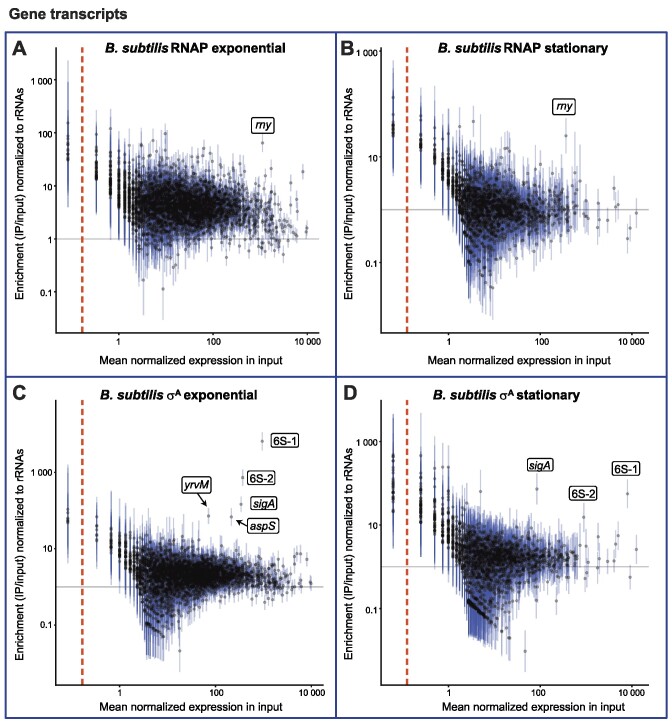
RIP-seq in *Bacillus subtilis*. Quantification of RIP-seq data for RNAP (**A, B**) and σ^A^ (**C, D**) for each annotated gene in *B. subtilis* in exponential and stationary phase. For intergenic regions, please see Supplementary Figure S10. For further details, see legend to Figure 1.

**Figure 10.**
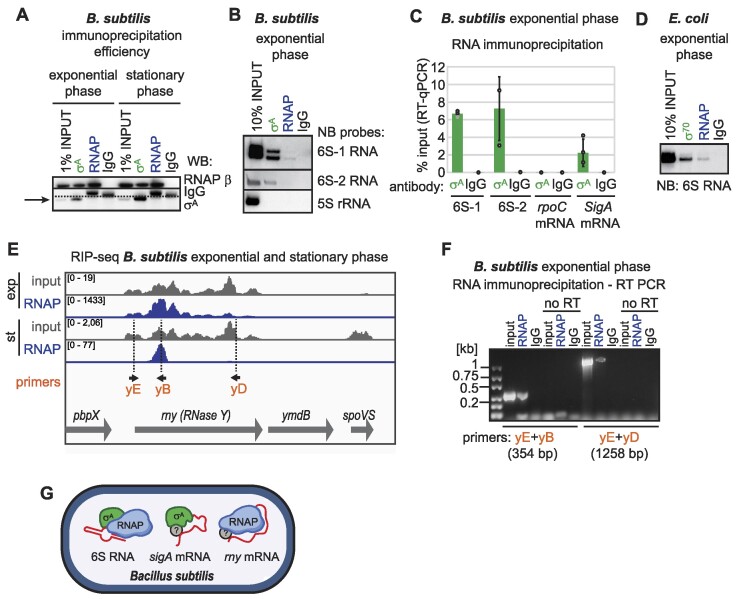
RNA interactions of σ^A^-RNAP, RNAP and σ^A^ in *B. subtilis*. (**A**) Amounts of immunoprecipitated σ^A^ and RNAP β subunit from *B. subtilis* exponential and stationary phase lysates examined by western blotting. The arrow indicates the position of σ^A^ bands. (**B**) The lysates from *B. subtilis* exponential phase cells were incubated with anti-σ^A^ and anti-RNAP antibodies, co-immunoprecipitated RNAs were isolated and the amounts of 6S-1, 6S-2 and 5S rRNA (negative control) detected by specific probes and northern blotting (**B**) or the level of co-immunoprecipitated 6S-1, 6S-2, *rpoC* and *sigA* mRNA was measured by RT-qPCR (**C**). (**D**) A lysate from *E. coli* mid-exponential phase was incubated with anti-σ^70^ and anti-RNAP antibodies, co-immunoprecipitated RNA isolated and the amount of 6S RNA detected by northern blotting. (**E**) Detailed view on RIP-seq mapped reads at the RNase Y (*rny*) locus and in flanking genes. RIP-seq was performed for exponential and stationary phase *B. subtilis* cells, the positions of primers used for RT-PCR validation are shown. (**F**) RT-PCR using RNA associated with RNAP in exponentially growing *B. subtillis* cells. RNAP binds intact *rny* mRNA in exponential phase. (**G**) Summary of RNAs that were detected by RIP-seq in *B. subtilis*. Question marks indicate that the exact composition of individual RNA-protein complexes is not known.

We performed RIP-seq with the anti-RNAP and the anti-σ^70^ antibody [also recognizing *B. subtilis* σ^A^, (32)] in exponential and stationary phases of *B. subtilis* BaSysBio strain 168 (Figure 9 shows annotated genes, Supplementary Figure S10 shows transcripts from intergenic regions). 6S-1 and 6S-2 RNAs were enriched on σ^A^ in stationary phase (Figure 9D). 6S-1 interacted with σ^A^-RNAP more strongly than 6S-2 RNA (55-fold enrichment versus 15-fold enrichment, respectively, Supplementary Table S10). Importantly, 6S-1 and 6S-2 RNA were highly enriched on σ^A^ also in exponential phase (Figure 9C). Again, the interaction with σ^A^-RNAP was more pronounced for 6S-1 than 6S-2 RNA (∼6500-fold enrichment versus ∼730-fold enrichment, respectively, Supplementary Table S10). 6S-1 and 6S-2 RNAs were more enriched on σ^A^ in exponential than in stationary phase (Figure 9C and D), although the amount of immunoprecipitated σ^A^ was comparable in both phases (Figure 10A).

We confirmed the 6S-1 and 6S-2 association with σ^A^-RNAP holoenzyme in exponential phase by northern blotting (Figure 10B) and RT-qPCR (Figure 10C). This is consistent with the previously published data showing the presence of 6S-1 and 6S-2 in the σ^A^-RNAP complex also in exponential phase (8) and the observed synthesis of pRNA derived from 6S-1 and 6S-2 in exponential phase (23). However, other data previously showed the association of 6S-1 and 6S-2 RNAs with RNAP mainly in stationary phase (86). Our data rather supports the presence of 6S-1/ and 6S-2/σ^A^-RNAP complexes also in exponential phase.

In addition, we showed that 6S RNA in *E. coli* (*ssrS*) also interacts with σ^70^-RNAP holoenzyme in exponential phase (Figure 10D). The anti-RNAP β antibody preferentially binds to the core of *B. subtilis* RNAP (Figure 10A). Consistently, we detected almost no 6S RNA signal in RNAP immunoprecipitations (Figure 10B and D) or *B. subtilis* RIP-seq data (Figure 9A and B) as 6S RNA does not bind to the RNAP core.

Besides 6S-1 and 6S-2 RNA, parts of *aspS* and *yrvM* transcripts were enriched in σ^A^ RIP-seq in exponential phase (Figure 9C). *aspS* and *yrvM* are the 5′ and 3′ flanking genes of *bsrA* (encoding 6S-1 RNA) showing that 6S-1 RNA can bind to the σ^A^-RNAP holoenzyme even when it is extended by mRNA sequences derived from the neighboring genes. In addition to 6S RNAs, the *sigA* transcript was bound to σ^A^ in RIP-seq from both growth phases (Figure 9C and D). *sigA* was enriched ∼150-fold in exponential and more than 70-fold in stationary phase (Supplementary Table S10). We cannot distinguish whether the *sigA* transcript binds to the free σ^A^ or σ^A^-RNAP holoenzyme (the anti-σ^70^ antibody immunoprecipitated also the σ^A^-RNAP complex, Figure 10A) but we confirmed the interaction of σ^A^ protein with its own *sigA* transcript by RT-qPCR (Figure 10C), indicating that the association of σ proteins with σ transcripts is common in different bacterial species (*M. smegmatis, M. tuberculosis, B. subtilis*).

### RNAP binds the RNase Y transcript in *Bacillus subtilis*

RIP-seq revealed an interaction between RNAP and mRNA encoding RNase Y (*rny* gene) (87) (Figures 9A, B and 10E). *rny* RNA was enriched ∼64-fold on RNAP in RIP-seq data from exponential phase (Supplementary Table S10). *rny* reads mapping to RNAP represented 0.29%, 0.68% and 0.27% of all sequenced RNAs in three biological replicates from exponential phase (Supplementary Table S10). This rather low association with RNAP indicates that *rny* RNA does not sequester RNAP but RNAP might rather regulate *rny* mRNA, which is further supported by the presence of full-length *rny* mRNA on RNAP (Figure 10F). Previously, it was shown that the expression of the *rny* gene is genetically linked to that of the *rpoB* and *rpoC* RNAP subunits (88) but the biological explanation of this observation is not yet known. Here we show that mRNA transcription and RNA degradation machinery are indirectly linked through *rny* mRNA that interacts with RNAP. Figure 10G summarizes RNAs that associate with the transcriptional machinery in *B. subtilis*.

To conclude, in addition to 6S or Ms1 RNAs, other RNAs associate with the bacterial transcription machinery; some of these interactions are species-specific (such as *rny* RNA–RNAP), but some seem to be evolutionarily conserved (such as σ^A^/σ^B^ binding to σ^A^/σ^B^ transcripts). Furthermore, other so far unknown RNAs associating with RNAP core or holoenzymes probably exist in the bacterial kingdom (such as CoRP RNA in corynebacteria).

## Discussion

### RIP-seq is a powerful tool for identification of RNAs interacting with the transcription machinery

We have established a native RIP-seq protocol to detect RNAs that associate with the transcriptional machinery—RNAP and the primary σ factors. Then, we designed a data analysis pipeline based on DESeq2, which allowed us to quantify the enrichment of individual transcripts either on RNAP or with the primary σ factor. We performed RIP-seq in several bacterial species in different growth phases to obtain a general view on RNAs interacting with the bacterial RNA polymerase. In each bacterial strain, we found at least one abundant RNA that has the potential to bind RNAP. We also show that regarding RNAP-interacting RNAs, bacterial species differ, and the regulatory mechanisms identified in *E. coli* or *B. subtilis* are not necessarily applicable to other bacteria.

### Abundant RNAs associated with the transcription machinery

**Figure 11.**
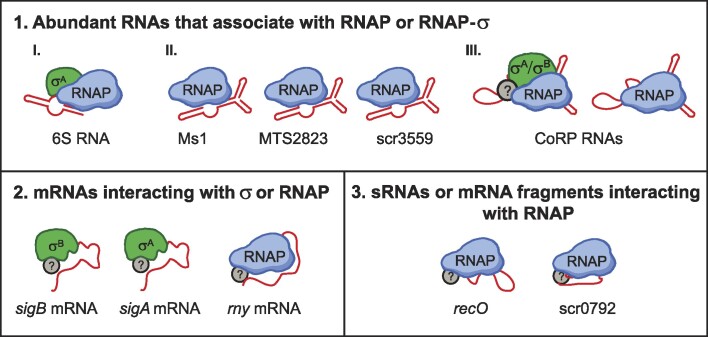
Overview of RNAs identified by RIP-seq. Group 1 represents abundant RNAs that associate with either RNAP (Ms1 homologs) or σ^A^-RNAP (6S RNAs) or both (CoRP RNAs). Group 2 includes mRNAs interacting with RNAP or σ factors. Group 3 contains sRNAs or fragments of mRNAs associated with RNAP. Question marks indicate that the exact composition of individual RNA-protein complexes is not known.

Abundant RNAP-associating RNAs can be divided into three classes: i. well-known and studied 6S RNAs which bind to RNAP in the complex with the primary σ factor (RIP-seq in *B. subtilis*); ii. Ms1 RNAs which bind the RNAP core (RIP-seq in *M. smegmatis, M. tuberculosis* and *S. coelicolor*); iii. CoRP RNAs which bind both RNAP core and RNAP holoenzyme in complex with the primary σ factor (RIP-seq in *C. glutamicum*) (Figure 11). In each bacterial species, there was only one type of abundant RNAP-associating RNA present. For example, we found only Ms1 and no 6S RNAs in *M. smegmatis, M. tuberculosis* and *S. coelicolor*. Contrarily, in *B. subtilis*, only 6S-1 and 6S-2 RNAs were detected, but no Ms1 homolog was identified by RIP-seq. We also assume that other classes of abundant RNAP-associating RNAs exist in bacteria. Previously, we found putative homologs of Ms1 in many actinobacterial species (37) but in some bacteria (such as corynebacteria) neither 6S RNA nor Ms1 was discovered with bioinformatic approaches searching for conserved gene synteny and secondary structure similarity. RIP-seq allowed us to detect these RNAs experimentally. Using the RIP-seq approach, we revealed CoRP RNA, a completely new type of abundant RNAP-associating RNA. We assume that for example in *Bifidobacterium bifidum* or in *Micrococcus luteus*, where no 6S RNA or Ms1 have been found (37), RIP-seq could identify unique types of abundant RNAP-associating RNAs. We propose that each bacterial species contains one type of abundant RNAP-associating RNA, but these RNAs might differ from the known 6S or Ms1 RNAs. Hence, they may have simply escaped identification by the currently used bioinformatic approaches.

6S and Ms1 RNAs are not essential (20,34,89–93) and represent an additional level of bacterial transcription regulation to fine-tune gene expression. In *B. subtilis* and *E. coli*, 6S RNAs associate with RNAP in complex with the primary σ factor also in exponential phase of growth (Figure 10). In *B. subtilis*, the 6S-1 and 6S-2 serve as templates for pRNA synthesis in both exponential and stationary phases (23), indicating the presence of 6S/σ^A^-RNAP complexes during the exponential phase as well. Therefore, 6S RNAs do not only sequester σ^A^/σ^70^–RNAP in stationary phase to decrease σ^A^/σ^70^–RNAP activity as proposed previously (9) but rather modulate gene expression during the entire bacterial growth. 6S RNAs thus enable bacteria to adjust the available levels of σ^A^/σ^70^–RNAP in all growth phases.

Abundant RNAP-associating RNAs remarkably differ in their primary sequences among various bacterial species and were probably evolutionarily optimized to bind RNAPs in their species-specific cellular context. We hypothesize that modified abundant RNAP-associating RNAs could be used as species-specific RNAP inhibitors in the future. These ‘RNA antibiotics’ could target only bacterial pathogens and not the other bacteria in the human microbiome.

### mRNAs and other RNAs associated with the transcription machinery

In addition to abundant RNAP-associating RNAs, RIP-seq revealed that both the primary σ factor and RNAP can interact with mRNAs (Figure 11). In several bacterial species (*B. subtilis*, *M. smegmatis* and *M. tuberculosis*), we observed interactions of σ^A^/σ^B^ with their own transcripts. In *B. subtilis*, RNAP associates with mRNA encoding RNase Y, suggesting a regulatory link between transcription and RNA degradation. We assume that these mRNAs do not sequester RNAP or σ^A^/σ^B^, but the binding of RNAP or σ^A^/σ^B^ to these mRNAs rather regulates mRNA stability or translation.

Other low abundant RNAP-associated RNAs (*recO*, scr0792 or ∼260 nt RNA in *M. tuberculosis*) could be a part of specific ribonucleoprotein complexes. These RNAs might regulate RNAP together with transcription factors, similar to what was shown for 7SK RNA and p-TEFB in eukaryotes (94–96). Mycobacteria contain many transcription factors with the WYL domain which is often found in RNA-binding proteins (97). Therefore, low abundant RNAs that associate with RNAP (such as *recO*) might play a role in activation of specific transcription factors. We assume that some protein complexes involved in the regulation of bacterial transcription could be in fact ribonucleoprotein complexes.

## Conclusions

In conclusion, bacteria include diverse sets of RNAP-associated RNAs that have different functions. In addition to abundant RNAP-associating RNAs, low expressed RNAs can also specifically interact with RNAP. Our data show that other, previously unidentified RNAs associate with RNAP or the primary σ factor in bacteria. These novel RNAs expand the possible mechanisms of bacterial transcription regulation. We hypothesize that 6S RNA or Ms1 RNA are only the tip of the iceberg – they were the first to be identified due to their high abundance, however, other lower expressed regulatory RNAs interacting with RNAP or the primary σ factor likely await their discovery.

## Supplementary Material

gkae081_Supplemental_Files

## Data Availability

Sequencing data are avaiable at ArrayExpress: *M. smegmatis* RIP-seq E-MTAB-11692, *B. subtillis* RIP-seq E-MTAB-11693, *S. coelicolor* RIP-seq E-MTAB-11694, *C. glutamicum* RIP-seq E-MTAB-12351* and E-MTAB-13608, *M. tuberculosis* RIP-seq E-MTAB-12350 and E-MTAB-13584, *M. smegmatis* ChIP-seq E-MTAB-12349. The mass spectrometry data have been deposited to the ProteomeXchange Consortium via the PRIDE partner repository with the dataset identifier PXD047705. All original code has been deposited at Zenodo under DOI: 10.5281/zenodo.10286942. *E-MTAB-12351 contains data that have not been used in this manuscript due to partial contamination by other bacterial species (tracks Cg_input_ex_1, Cg_input_st_1, Cg_RNAP_ex_1, Cg_RNAP_st_1, Cg_sigA_ex_1, Cg_sigA_st_1).
