# Supplementary material for: RIP-seq reveals RNAs that interact with RNA polymerase and primary sigma factors in bacteria
Source: Nucleic Acids Res. 2024 Feb 13;52(8):4604–26. doi: 10.1093/nar/gkae081 (PMC11077062; doi:10.1093/nar/gkae081)
Supplement: gkae081_Supplemental_Files [file gkae081_supplemental_files.zip › Supplementary data and figures.pdf]

RIP-seq reveals RNAs that interact with RNA polymerase and primary sigma factors in bacteria

SUPPLEMENTARY TABLES

Supplementary Table 1 shows *M. smegmatis* RIP-seq read counts from exponential („EX“) or stationary („ST“) phase of growth. „Anti-RNAP“ and „anti-sigma“ are read counts from the RNA libraries co-immunoprecipitated with RNA polymerase or with σ^A/σ^B. Reads mapping to the intergenic regions are in „counts_intergenic“. RIP-seq fold-change („log2FoldChange“) and FDR-corrected p-value („padj“) for each *M. smegmatis* gene or intergenic region are shown. „InputMean“ is the mean number of reads in inputs. „lfcSE“ is the standard error of log2FoldChange.

Supplementary Table 2 *M. smegmatis* ChIP-seq peaks in stationary phase of growth. „ppval“ represents the significance of the ChIP-seq peak.

Supplementary Table 3 *M. smegmatis* proteins present in anti-σ^{70} antibody immunoprecipitates from the stationary phase, including their Mascot Protein Scores. These proteins were identified in two biological replicates and were absent in IgG control immunoprecipitates. Protein Scores were calculated by Mascot software, for details and the complete list of identified proteins, see PRIDE repository entry with the dataset identifier PXD047705.

Supplementary Table 4 shows *M. tuberculosis* RIP-seq read counts from stationary phase of growth. „Anti-RNAP“ and „anti-sigma“ are read counts from the RNA libraries co-immunoprecipitated with RNA polymerase or with σ^A. Reads mapping to the intergenic regions are in „counts_intergenic“. RIP-seq fold-change („log2FoldChange“) and FDR-corrected p-value („padj“) for each *M. tuberculosis* gene or intergenic region are shown. „InputMean“ is the mean number of reads in inputs. „lfcSE“ is the standard error of log2FoldChange.

Supplementary Table 5 shows *S. coelicolor* RIP-seq read counts from exponential – 42 hours post-germination („42“) or stationary – 66 hours post-germination („66“) phase of growth. „Anti-RNAP“ and „anti-HrdB“ are read counts from the RNA libraries co-immunoprecipitated with RNA polymerase or with HrdB-HA. Reads mapping to the intergenic regions are in „counts_intergenic“. RIP-seq fold-change („log2FoldChange“) and FDR-corrected p-value

(„padj“) for each *S. coelicolor* gene or intergenic region are shown. „InputMean“ is the mean number of reads in inputs. „lfcSE“ is the standard error of log2FoldChange.

Supplementary Table 6 *C. glutamicum* proteins identified in anti-σ^{70} antibody immunoprecipitates from the stationary phase. Fifteen proteins with the highest Mascot Protein Scores, absent in IgG control immunoprecipitates, are shown. Protein Scores were calculated by Mascot software, for details and the complete list of identified proteins, see PRIDE repository entry with the dataset identifier PXD047705.

Supplementary Table 7 *C. glutamicum* proteins identified in anti-RNAP antibody immunoprecipitates from the stationary phase. Fifteen proteins with the highest Mascot Protein Scores, absent in IgG control immunoprecipitates, are shown. Protein Scores were calculated by Mascot software, for details and the complete list of identified proteins, see PRIDE repository entry with the dataset identifier PXD047705.

Supplementary Table 8 shows *C. glutamicum* RIP-seq read counts from exponential („EX“) or stationary („ST“) phase of growth. „Anti-RNAP“ and „anti-sigma“ are read counts from the RNA libraries co-immunoprecipitated with RNA polymerase or with σ^A/σ^B. Reads mapping to the intergenic regions are in „counts_intergenic“. RIP-seq fold-change („log2FoldChange“) and FDR-corrected p-value („padj“) for each *C. glutamicum* gene or intergenic region are shown. „InputMean“ is the mean number of reads in inputs. „lfcSE“ is the standard error of log2FoldChange.

Supplementary Table 9 Summary of BLASTn searches for CoRP RNA sequences in *Corynebacterium* genus. A total of 313 high scoring pairs (HSPs) were found by BLAST within *Corynebacterium* genus in two separate searches (the first search was within *Corynebacterium glutamicum*, the second search was done in *Corynebacterium* genus with *C. glutamicum* excluded from the search). The HSPs were the filtered based on these criteria:
1) sequence containing HSP must have tRNA genes annotated
2) the HSP must lie between tRNA Gln - tRNA Glu pair and tRNA-Glu. The distance of tRNA Gln - tRNA Glu must be < 50 and the distance of tRNA Gln - tRNA Glu pair and tRNA-Glu < 10,000 bps, respectively.
The filtering resulted in 295 HSPs, with the most HSPs (14) excluded due to the missing annotation. Sequences very similar (E-value < 1E-100) to CoRP RNA were found in several corynebacterial species, HSPs with rather low E-value (> 1E-6) are supported with the genome context of tRNA genes, which appears to be conserved in majority of genomes despite of the

large variability in the region length, in the number of annotated proteins and in the protein annotations within the region.

The variability of this region is unexpected and will be further investigated.

Supplementary Table 10 shows *B. subtilis* RIP-seq read counts from exponential („EX“) or stationary („ST“) phase of growth. „Anti-RNAP“ and „anti-sigma“ are read counts from the RNA libraries co-immunoprecipitated with RNA polymerase or with σ^A. Reads mapping to the intergenic regions are in „counts_intergenic“. RIP-seq fold-change („log2FoldChange“) and FDR-corrected p-value („padj“) for each *B. subtilis* gene or intergenic region are shown. „InputMean“ is the mean number of reads in inputs. „lfcSE“ is the standard error of log2FoldChange.

Supplementary Table 3

**Protein accession number**	**Protein description**	**Protein Score in replica 1**	**Protein Score in replica 2**
P60281|RPOB_MYCS2	DNA-directed RNA polymerase subunit beta	2097	18364
A0QSL8|RPOA_MYCS2	DNA-directed RNA polymerase subunit alpha	463	7490
A0R218|A0R218_MYCS2	Transcription termination factor Rho	98	4287
A0QS66|RPOC_MYCS2	DNA-directed RNA polymerase subunit beta´	5682	24315
A0QSP8|RL13_MYCS2	Large ribosomal subunit protein uL13	137	517
A0QW02|A0QW02_MYCS2	RNA polymerase sigma factor SigA	3485	10631
A0QVZ5|A0QVZ5_MYCS2	RNA polymerase sigma factor SigB	1994	5605
A0QZ11|RBPA_MYCS2	RNA polymerase-binding protein RbpA	1729	4207
A0R3I5|A0R3I5_MYCS2	Molybdopterin biosynthesis protein *	1453	2783
A0QSG0|RL24_MYCS2	Large ribosomal subunit protein uL24	91	342
A0QTD8|A0QTD8_MYCS2	RNA polymerase sigma factor SigF	65	311

* This protein is subject of a manuscript in revision.

Supplementary Table 6

**Protein accession number**	**Protein description**	**Protein Score**
Q8NT25|RPOC_CORGL	DNA-directed RNA polymerase subunit beta´	29172
Q8NT26|RPOB_CORGL	DNA-directed RNA polymerase subunit beta	16968
Q8NPA3|Q8NPA3_CORGL	RNA polymerase sigma factor SigA	11256
Q8NSV2|RPOA_CORGL	DNA-directed RNA polymerase subunit alpha	10490
Q8NS09|Q8NS09_CORGL	Molybdopterin biosynthesis enzymes	5314
Q8NQF8|Q8NQF8_CORGL	RNA polymerase-binding protein RbpA	4111
Q79VF3|Q79VF3_CORGL	RNA polymerase sigma factor SigB	3743

Q8NLS2|Q8NLS2_CORGL	NYN domain-containing protein	3738
Q8NQR5|Q8NQR5_CORGL	Ribosomal protein S1	3406
Q8NP40|IF2_CORGL	Translation initiation factor IF-2	2585
Q8NP01|RS2_CORGL	Small ribosomal subunit protein uS2	2214
Q8NT01|RS3_CORGL	Small ribosomal subunit protein uS3	1974
Q8NSX5|RS5_CORGL	Small ribosomal subunit protein uS5	1581
Q8NSV4|RS4_CORGL	Small ribosomal subunit protein uS4	1575
Q8NQ43|RPOZ_CORGL	DNA-directed RNA polymerase subunit omega	1425

Supplementary Table 7

**Protein accession number**	**Protein description**	**Protein Score**
Q8NT25|RPOC_CORGL	DNA-directed RNA polymerase subunit beta′	47284
Q8NT26|RPOB_CORGL	DNA-directed RNA polymerase subunit beta	37110
Q8NSV2|RPOA_CORGL	DNA-directed RNA polymerase subunit alpha	18918
Q8NT43|Q8NT43_CORGL	2-succinyl-5-enolpyruvyl-6-hydroxy-3-cyclohexene-1-carboxylate synthase	4421
Q8NPA3|Q8NPA3_CORGL	RNA polymerase sigma factor SigA	3604
Q8NQ44|Q8NQ44_CORGL	Coenzyme A biosynthesis bifunctional protein CoaBC	3181
Q8NQQ3|Q8NQQ3_CORGL	UvrD-like helicase ATP-binding domain-containing protein	2518
Q8NQ43|RPOZ_CORGL	DNA-directed RNA polymerase subunit omega	2124
Q8NQR5|Q8NQR5_CORGL	Ribosomal protein S1	2081
Q8NND3|GLMS_CORGL	Glutamine--fructose-6-phosphate aminotransferase	1280
Q8NP01|RS2_CORGL	Small ribosomal subunit protein uS2	1188
Q8NU48|Q8NU48_CORGL	NAD-dependent aldehyde dehydrogenases	1128
Q8NP89|Q8NP89_CORGL	HrpA-like helicases	1122
Q8NP40|IF2_CORGL	Translation initiation factor IF-2	1101
Q8NQF8|Q8NQF8_CORGL	RNA polymerase-binding protein RbpA	1083

Supplementary Table 9 Summary of BLASTn searches for CoRP RNA sequences in *Corynebacterium* genus.

**Species group***	**n HSPs**	**min E-val**	**max E-val**	**tRNA-Glu intergenic region length (min - max)**	**n annotated proteins (min - max)**
[Brevibacterium] flavum	3	0.00E+00	0.00E+00	1550 - 2284	0 - 2
Corynebacterium suranareeae	1	0.00E+00	0.00E+00	817 - 817	0 - 0
Corynebacterium glutamicum	32	0.00E+00	4.66E-170	801 - 2284	0 - 3
Corynebacterium crudilactis	1	8.87E-159	8.87E-159	955 - 955	0 - 0
Corynebacterium deserti	1	9.48E-150	9.48E-150	1519 - 1519	1 - 1
Corynebacterium callunae	1	5.68E-130	5.68E-130	814 - 814	0 - 0
Corynebacterium efficiens	1	1.35E-101	1.35E-101	795 - 795	1 - 1

Corynebacterium occultum	1	1.53E-40	1.53E-40	773 - 773	0 - 0
Corynebacterium comes	1	4.70E-22	4.70E-22	708 - 708	0 - 0
Corynebacterium humireducens	1	3.41E-21	3.41E-21	699 - 699	0 - 0
Corynebacterium halotolerans	1	4.81E-19	4.81E-19	711 - 711	0 - 0
Corynebacterium marinum	1	1.29E-18	1.29E-18	705 - 705	0 - 0
Corynebacterium testudinoris	1	4.92E-16	4.92E-16	713 - 713	0 - 0
Corynebacterium sp.	4	2.58E-14	5.50E-01	707 - 4500	0 - 4
Corynebacterium maris	1	3.72E-09	3.72E-09	756 - 756	0 - 0
Corynebacterium vitaeruminis	1	1.02E-05	1.02E-05	791 - 791	0 - 0
Corynebacterium frankenforstense	1	7.42E-05	7.42E-05	1942 - 1942	1 - 1
Corynebacterium pseudotuberculosis	145	5.37E-04	1.05E-02	2356 - 2363	1 - 4
Corynebacterium yudongzhengii	1	1.05E-02	1.05E-02	7803 - 7803	5 - 5
Corynebacterium ulcerans	20	1.05E-02	7.59E-02	2370 - 2411	1 - 3
Corynebacterium hindlerae	1	2.82E-02	2.82E-02	6852 - 6852	4 - 4
Corynebacterium silvaticum	8	2.82E-02	2.82E-02	2373 - 2373	1 - 1
Corynebacterium diphtheriae	65	7.59E-02	5.50E-01	2836 - 8987	2 - 12
Corynebacterium epidermidicanis	1	7.59E-02	7.59E-02	5295 - 5295	4 - 4
Corynebacterium rouxii	1	5.50E-01	5.50E-01	2831 - 2831	3 - 3

* As reported in GenBank.

SUPPLEMENTARY MATERIALS AND METHODS

Oligonucleotides

**Primers**		
name	Sequence 5´ to 3´	usage
Mtb_MTS2823_F	CCAAGGCTCGATCCAGAAGA	RT-qPCR
Mtb_MTS2823_R	TGTTCGCAATTACGCAGACC	RT-qPCR
Mtb_23SrRNA_rrl_F	TCAACCATAGACTCGGCGAA	RT-qPCR
Mtb_23SrRNA_rrl_R	AGTTGTAGTGAAGGTCCCGG	RT-qPCR
Msmeg_rpoB_F	AGGGCACCTTCATCATCAAC	RT-qPCR
Msmeg_rpoB_R	GGTGGACTTGTCGATGGTCT	RT-qPCR
Msmeg_sigB_M1	TCGCTTTCGTTGTCGCATC	RT-PCR
Msmeg_sigB_M2	CCGCAACGGCGAGCGTGCCG	RT-PCR
Msmeg_sigB_M3	GGTATCCCGGTCGAGAAGAT	RT-PCR
Msmeg_sigB_M4	GCTCGACCTGATCCAGGAG	RT-PCR
Msmeg_sigB_F	GGCGACTTCATCGAGGATT	RT-qPCR
Msmeg_sigB_R	CGAGCACGTAACGGATGTC	RT-qPCR
Msmeg_recO_rA	GGCCGCAATCTCGACATC	RT-qPCR, RT-PCR
Msmeg_recO_rC	GTACCGCCCGTAGTCACTG	RT-qPCR, RT-PCR
Msmeg_recO_rB	GTACCCGCAGCAAGTTCG	RT-PCR
Msmeg_recO_rD	GCCATATCCTGCCTGACCA	RT-PCR

Msmeg_sigA_F	GATCGGCCAGGTCTACGG	RT-qPCR
Msmeg_sigA_R	TAGTCCAGGTAGTCGCGCAG	RT-qPCR
Msmeg_sigF_F	GATCTCGACATGGGCCTCG	RT-qPCR
Msmeg_sigF_R	CGATCTGGGTCTGCGTCAT	RT-qPCR
Msmeg_rplM_F	GGCCGTCAGATCCAGAAGAA	RT-qPCR
Msmeg_rplM_R	CACCTGCTTGATCTCGAACG	RT-qPCR
Msmeg_hsp20_F	ATGAGCACGCTGATGAAGAC	RT-qPCR
Msmeg_hsp20_R	ATCCCTTGAACCAGTCGTCG	RT-qPCR
Cg_CoRP_C1	GTTCATAGCCGATCACCAACA	RT-PCR, 3´ RACE
Cg_CoRP_C2	TTCGAATGAAGGTGTCTGGG	RT-PCR, 5´ RACE
Cg_CoRP_C3	CCGAGTGTGGGTTGTTGAAG	5´ RACE
Cg_CoRP_C4	CAGGTTGAAGCCACTGTGAG	3´ RACE
Cg_CoRP_iv_F	AACAAGCTTTAATACGACTCACTATAGATACAGAGAAGTTCATAGCCG	Cloning of CoRP construct
Cg_CoRP_iv_R	TACTCTAGAAGGCTAGGTCTCCCTGTAACGACACCCTCTTTCGA	Cloning of CoRP construct
Bsu_rny_yE	GGAGGTGAAAGTATGACCCCA	RT-PCR
Bsu_rny_yB	CTTCTCCAACATCGCTTCCC	RT-PCR
Bsu_rny_yD	TAATGGAAGTCGGCTCCTCG	RT-PCR
Bsu_sigA_F	GATGACGGCCGTACAAGAAC	RT-qPCR
Bsu_sigA_R	ACGTTTACTTCTGCTAGGATGTC	RT-qPCR
Bsu_rpoC_F	CCGGACGTTCTGTAATCGTT	RT-qPCR
Bsu_rpoC_R	TGCGTTTCGCACTCTTAATG	RT-qPCR
Bsu_6S-1_F	ATGGCGTACATGCCTCTTTT	RT-qPCR
Bsu_6S-1_R	AATAGTGCCGTTGCAGCTTT	RT-qPCR
Bsu_6S-2_F	ATATCGAAGCCGGAATGTCA	RT-qPCR
Bsu_6S-2_R	CGTCTTGCCGTATGCAAGTAA	RT-qPCR
Plat_Spike_F	TTACTGCAGCTGAGGTCACA	RT-qPCR
Plat_Spike_R	AGCTCACACTCTGTCCAGTC	RT-qPCR
**Probes**		
Mtb_MTS2823_NB	Btn-ACCCAAAGGGCCGTCGATGCCATCT	Northern blot
Msmeg_recO_NB	Btn-GTGTACCGCCCGTAGTCACTGACGA	Northern blot
Sco_scr0792_1_NB	Btn-GATCGTCCCTGTGGGCAATAGGTGG	Northern blot
Sco_scr0792_2_NB	Btn-CGGCGAAAGGCTCCACAGGGCTTCA	Northern blot
Cg_CoRP_5´_NB	Btn-CGAGCTCCTGATTCACGCATCTCTA	Northern blot
Cg_CoRP_3´_NB	Btn-GCTTCAACCTGCTGCCACCGCTTTC	Northern blot
Bsu_6S1_NB	Btn-CTTGTAAGTCCTCTTTACCGAATGAAA	Northern blot
Bsu_6S2_NB	Btn-TCTGCAATGGAGGCATGACATTCCG	Northern blot
Bsu_5SrRNA_NB	Btn-GTATGGGAACGGGTGTGACCTCTTC	Northern blot
Eco_6S_NB	Btn-GTTCAAGGTGAATGTGTCGTCGCAG	Northern blot

sequence of *Plat* mRNA (718 bp)

pJET_*Plat* RNA transcript

GGGAGAGCGGCCGCAUGGGCAAGAGUUACACAGCGUGGAGGACCAACUCCCAG
GCACUCGGCCUGGGCAGACACAAUUAUUGUCGGAAUCCAGAUGGUGAUGCCAG
ACCUUGGUGCCAUGUGAUGAAGGACCGAAAGCUGACGUGGGAAUACUGUGACA
UGUCCCCAUGCUCCACCUGUGGCCUGAGGCAGUACAAACGGCCUCAGUUUAGA
AUUAAAGGAGGACUCUACACAGACAUCACCUCACACCCUUGGCAGGCUGCCAU
CUUUGUCAAGAACAAGAGGUCUCCUGGAGAGAGAUUCCUUUGUGGAGGGGUGC
UGAUCAGUUCCUGCUGGGUGCUGUCAGCUGCCCACUGCUUUCUAGAGAGGUUC
CCCCCCAAUCAUCUUAAAGUGGUCUUGGGCAGAACAUACAGGGUGGUCCCCGG
AGAGGAAGAACAGACAUUUGAGAUUGAAAAAUACAUAGUCCAUGAGGAAUUU
GAUGACGAUACUUAUGACAACGACAUCGCAUUACUGCAGCUGAGGUCACAGUC
CAAGCAAUGUGCCCAAGAGAGCAGCUCUGUUGGCACUGCCUGCCUCCCUGACC
CCAACCUGCAGCUCCCUGACUGGACAGAGUGUGAGCUUUCUGGCUACGGCAAG
CAUGAGGCAUCGUCUCCAUUCUUCUCUGCGGCCGCAAUCUUUCUAGAAGAUCU
CCUACAAUAUUCUCAGCUGCCAUGGAAAAU

SUPPLEMENTARY REFERENCES

1. Šiková, M., Janoušková, M., Ramaniuk, O., Páleníková, P., Pospíšil, J., Bartl, P., Suder, A., Pajer, P., Kubičková, P., Pavliš, O. *et al.* (2019) Ms1 RNA increases the amount of RNA polymerase in *Mycobacterium smegmatis*. *Mol Microbiol*, **111**, 354-372.
2. Gruber, A.R., Lorenz, R., Bernhart, S.H., Neuböck, R. and Hofacker, I.L. (2008) The Vienna RNA websuite. *Nucleic Acids Res*, **36**, W70-74.
3. Ibraim, I.C., Parise, M.T.D., Parise, D., Sfeir, M.Z.T., de Paula Castro, T.L., Wattam, A.R., Ghosh, P., Barh, D., Souza, E.M., Góes-Neto, A. *et al.* (2019) Transcriptome profile of *Corynebacterium pseudotuberculosis* in response to iron limitation. *BMC Genomics*, **20**, 663.
4. Luong, T.T., Nguyen, M.T., Chen, Y.W., Chang, C., Lee, J.H., Wittchen, M., Ton-That, H., Cruz, M., Garsin, D.A., Das, A. *et al.* (2021) Ribonuclease J-Mediated mRNA Turnover Modulates Cell Shape, Metabolism and Virulence in. *Microorganisms*, **9**.
5. Patel, H., Beber, M.E., Joshi, E., Han, D.W., Bot, N.-C., Manning, J., Yates, J.A.F., Talbot, A., Espinosa-Carrasco, J., Ewels, P. *et al.* (2023). 1.10.0 ed. Zenodo.
6. Patel, H., Ewels, P., Peltzer, A., Hammarén, R., Botvinnik, O., Sturm, G., Moreno, D., Vemuri, P., Silviamorins, Pantano, L. *et al.* (2021). 3.4 ed. Zenodo.

Supplementary Figure S1

```
MSMEG_2752      ----------------------------MANATTSRVDTDLDAQSPAADLVRVYLNGIGK      32
Rv2710          ------------------------MADAPTRATTSRVDSDLDAQSPAADLVRVYLNGIGK      36
MSMEG_2758      TAKAADDDIDEPSEKDKASGDFVWDEEESEALRQARKDA---ELTASADSVRAYLKQIGK      175
Rv2703          QTADDDEEIAEPTEKDKASGDFVWDEDESEALRQARKDA---ELTASADSVRAYLKQIGK      237
                                                  :* *:     : :** **.**: ***

MSMEG_2752      TALLNAADEVELAKRIEAGLYAQHLLDTK----KRLGEARKRDLQAVVRDGEAARRHLLE      88
Rv2710          TALLNAAGEVELAKRIEAGLYAEHLLETR----KRLGENRKRDLAAVVRDGEAARRHLLE      92
MSMEG_2758      VALLNAEEEVELAKRIEAGLYATQKLAELAEKGEKLPVQQRRDMQWICRDGDRAKNHLLE      235
Rv2703          VALLNAEEEVELAKRIEAGLYATQLMTELSERGEKLPAAQRRDMMWICRDGDRAKNHLLE      297
                .*****  ************** : :       ::*   ::**:  : ***: *:.****

MSMEG_2752      ANLRLVVSLAKRYTGRGMPLLDLIQEGNLGLIRAMEKFDYTKGFKFSTYATWWIRQAITR      148
Rv2710          ANLRLVVSLAKRYTGRGMPLLDLIQEGNLGLIRAMEKFDYTKGFKFSTYATWWIRQAITR      152
MSMEG_2758      ANLRLVVSLAKRYTGRGMAFLDLIQEGNLGLIRAVEKFDYTKGYKFSTYATWWIRQAITR      295
Rv2703          ANLRLVVSLAKRYTGRGMAFLDLIQEGNLGLIRAVEKFDYTKGYKFSTYATWWIRQAITR      357
                ****************** :**************:********:****************

MSMEG_2752      GMADQSRTIRLPVHLVEQVNKLARIKREMHQNLGREATDEELAEESGIPVEKINDLLEHS      208
Rv2710          GMADQSRTIRLPVHLVEQVNKLARIKREMHQHLGREATDEELAAESGIPIDKINDLLEHS      212
MSMEG_2758      AMADQARTIRIPVHMVEVINKLGRIQRELLQDLGREPTPEELAKEMDITPEKVLEIQQYA      355
Rv2703          AMADQARTIRIPVHMVEVINKLGRIQRELLQDLGREPTPEELAKEMDITPEKVLEIQQYA      417
                .****:****:***:** :***.**:**: *.**** * **** * .*  :*: :: :::

MSMEG_2752      RDPVSLDMPVGTDEEAPLGDFIEDSEAMSAENAVISELLHTDIRYVLATLDEREQQVIRL      268
Rv2710          RDPVSLDMPVGSEEEAPLGDFIEDAEAMSAENAVIAELLHTDIRSVLATLDEREHQVIRL      272
MSMEG_2758      REPISLDQTIGDEGDSQLGDFIEDSEAVVAVDAVSFTLLQDQLQSVLETLSEREAGVVRL      415
Rv2703          REPISLDQTIGDEGDSQLGDFIEDSEAVVAVDAVSFTLLQDQLQSVLDTLSEREAGVVRL      477
                *:*:***  :* : :: *******:**: * :**   **: ::: ** **.***  *:**

MSMEG_2752      RFGLDDGQPRTLDQIGKLFGLSRERVRQIEREVMAKLRNGERADRLRSYAS 319
Rv2710          RFGLDDGQPRTLDQIGKLFGLSRERVRQIERDVMSKLRHGERADRLRSYAS 323
MSMEG_2758      RFGLTDGQPRTLDEIGQVYGVTRERIRQIESKTMSKLRHPSRSQVLRDYLD 466
Rv2703          RFGLTDGQPRTLDEIGQVYGVTRERIRQIESKTMSKLRHPSRSQVLRDYLD 528
                **** ********:**:::*::***:**** ..*:***: .*:: **.* .
```

LQEMGREPTPEELAER *E. coli* peptide used for antibody production

Supplementary Figure S1. Amino acid sequences of σ^A and σ^B from *M. smegmatis* and *M. tuberculosis*. In *M. smegmatis*, *MSMEG_2752* and *MSMEG_2758* correspond to σ^B and σ^A, respectively. In *M. tuberculosis*, *Rv2710* and *Rv2703* correspond to σ^B and σ^A, respectively.
The N-terminal 118 aminoacids of *MSMEG_2758* and the N-terminal 180 aminoacids of *Rv2703* were omitted from the alignment.

Supplemenatary Figure S2

Intergenic regions transcripts

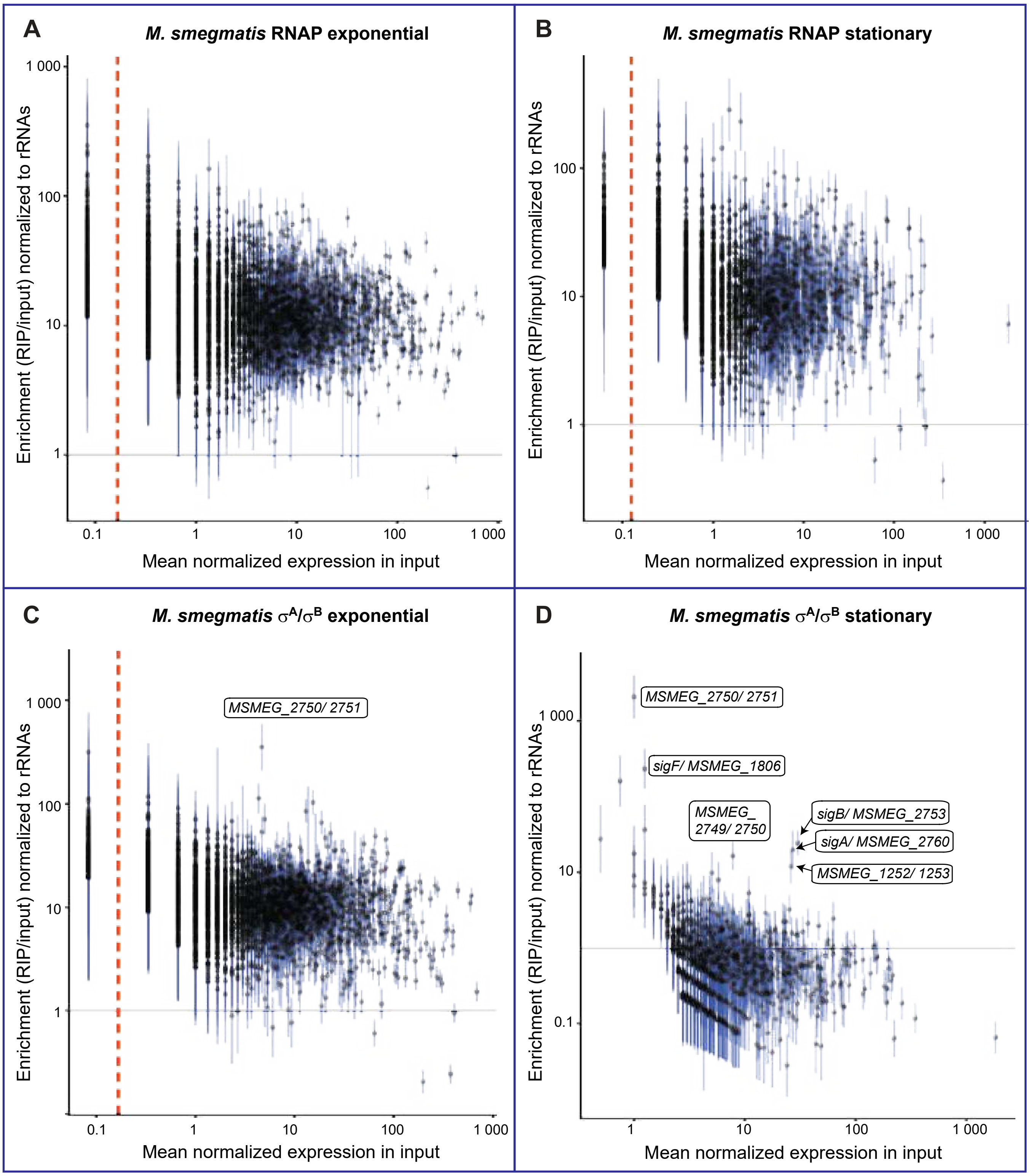

Supplementary Figure S2. Quantification of RIP-seq data in *M. smegmatis* in exponential and stationary phase for RNAP (**A, B**) and σ^A/σ^B (**C, D**) for each intergenic region in the genome. The horizontal axis (log scale) represents the mean of the normalized counts in the input, vertical axis (log scale) shows the estimated fold change in the ratio of read counts in immunoprecipitated to input samples, each normalized to the read counts for rRNA. Estimates (points) and 95 % confidence intervals (lines) are shown for all transcripts. Transcripts to the left of the vertical dashed line had zero reads in all replicates of the input material. The horizontal gray line marks a fold change of 1, i. e. no enrichment or depletion of the respective RNA transcript after immunoprecipitation.

Supplementary Figure S3

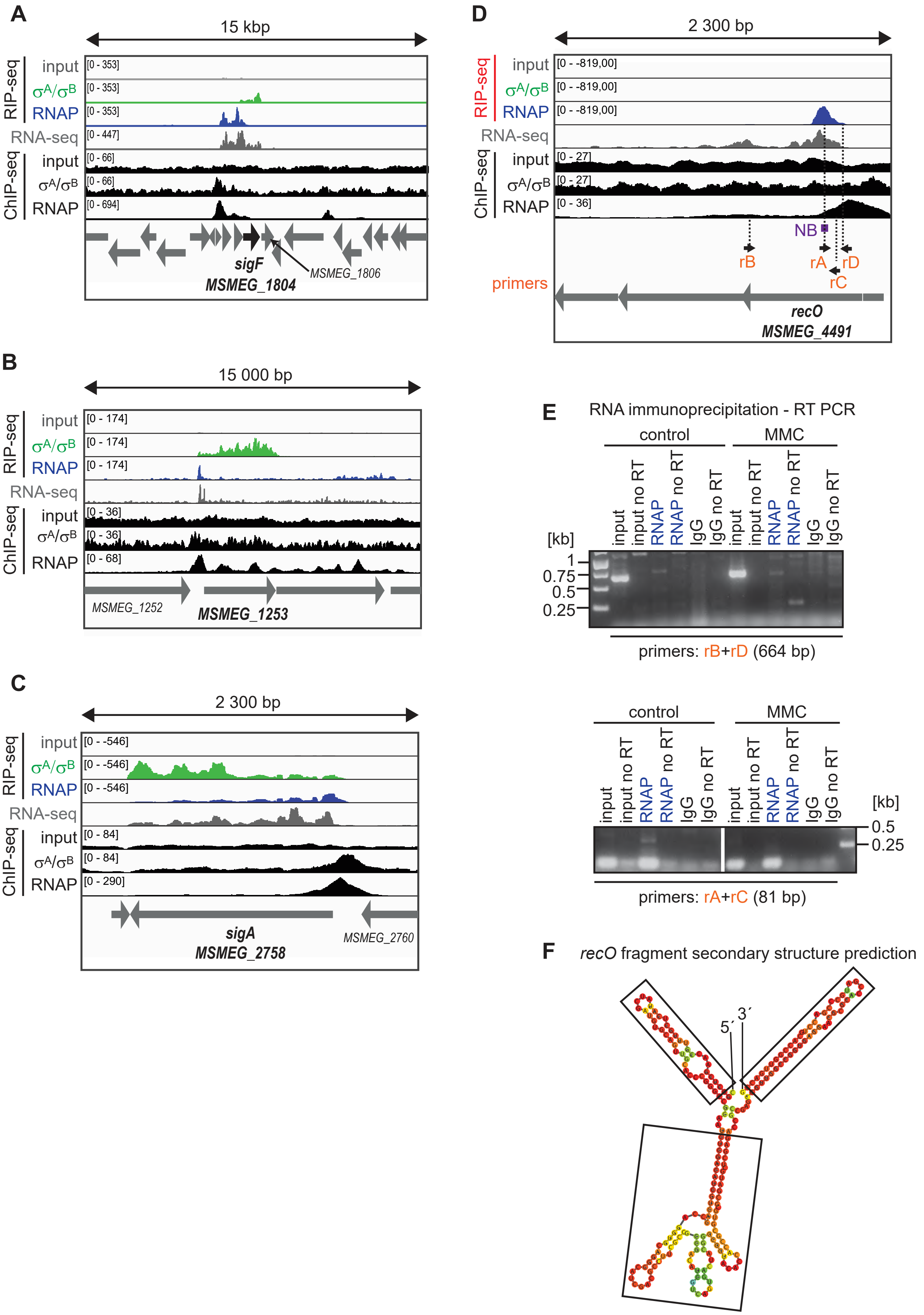

Supplementary Figure S3. A, B, C Stationary phase RIP-seq and ChIP-seq data from *M. smegmatis* for *sigF*, *MSMEG_1253* and *sigA* genes. RNA-seq data were published previously (1). The exact positions of RIP-seq peaks along the genes differ from the positions of the ChIP-seq peaks, indicating that free and not co-transcriptionally associated RNAs are bound to σ^A/σ^B. **D.** Stationary phase RIP-seq and ChIP-seq data from *M. smegmatis* for *recO* gene. RNA-seq data were published previously (1). The position of primers used in RT-PCR in **E** are shown. **E.** RT-PCR from RNA isolated from *M. smegmatis* control and mitomycin C (MMC) treated cells with different primers complementary to *recO* mRNA. Only the shortest amplicon (81 bp) was amplified from cDNA that was reverse transcribed from RNA associated with RNAP. A longer amplicon (664 bp) was not detected. **F.** Minimum free energy secondary structure prediction of the RIP-seq enriched fragment of *recO* mRNA from *M. smegmatis* was generated using RNAfold (Vienna RNAfold webserver(2)). The parts of the secondary structure with the highest base pair probabilities are highlighted in red. The locally stable secondary structures detected by RNALfold are marked by rectangles. The genome coordinates of the RIP-seq enriched fragment are NC_008596.1: 4,572,152-4,572,359.

Supplementary Figure S4

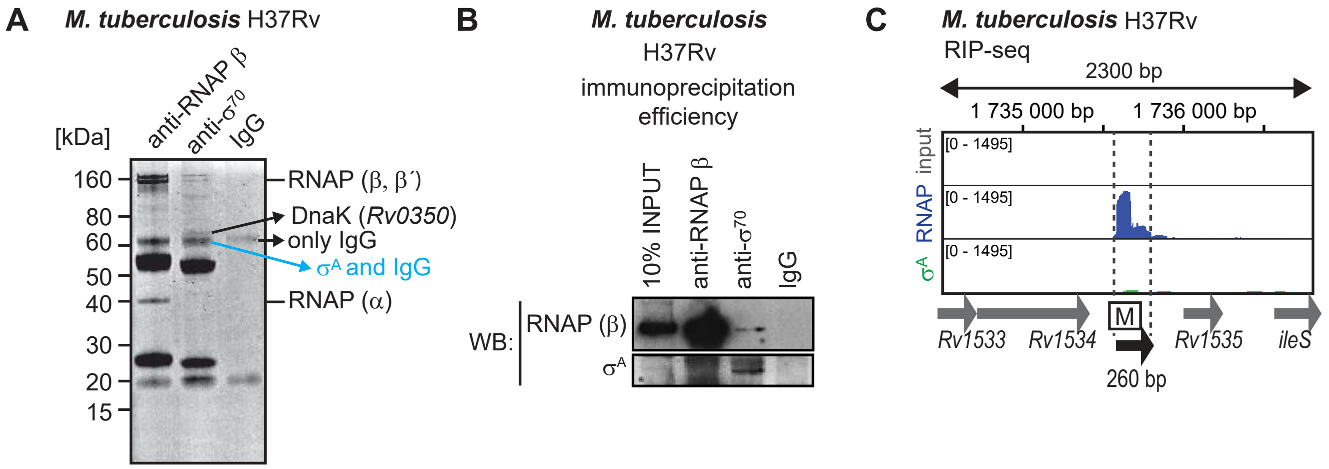

Intergenic regions transcripts

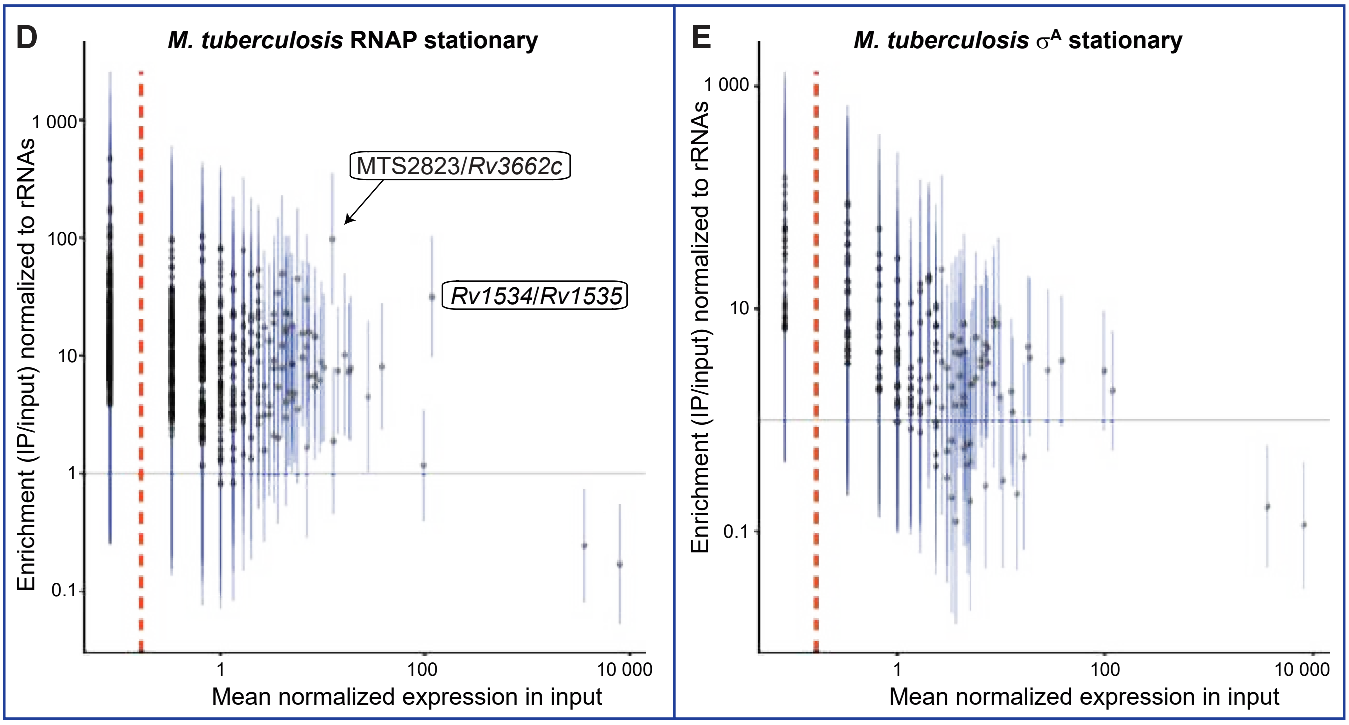

Supplementary Figure S4. **A.** Coomassie-stained SDS PAGE showing the proteins immunoprecipitated with anti-RNAP and anti-σ^{70} antibodies in *M. tuberculosis*. Anti-σ^{70} antibody immunoprecipitated σ^A and σ^A-RNAP holoenzyme. **B.** Western blotting of proteins immunoprecipitated with anti-RNAP and anti-σ^{70} antibodies in *M. tuberculosis*. **C.** RNA derived from the 5´end of *Rv1535* gene binds to RNAP in RIP-seq, but is not significantly enriched compared to other *M. tuberculosis* transcripts. Quantification of RIP-seq data in *M. tuberculosis* in stationary phase for RNAP (**D**) and σ^A (**E**) for each intergenic region in the genome. For further details, see legend to Figure S2.

Supplementary Figure S5

Intergenic region transcripts

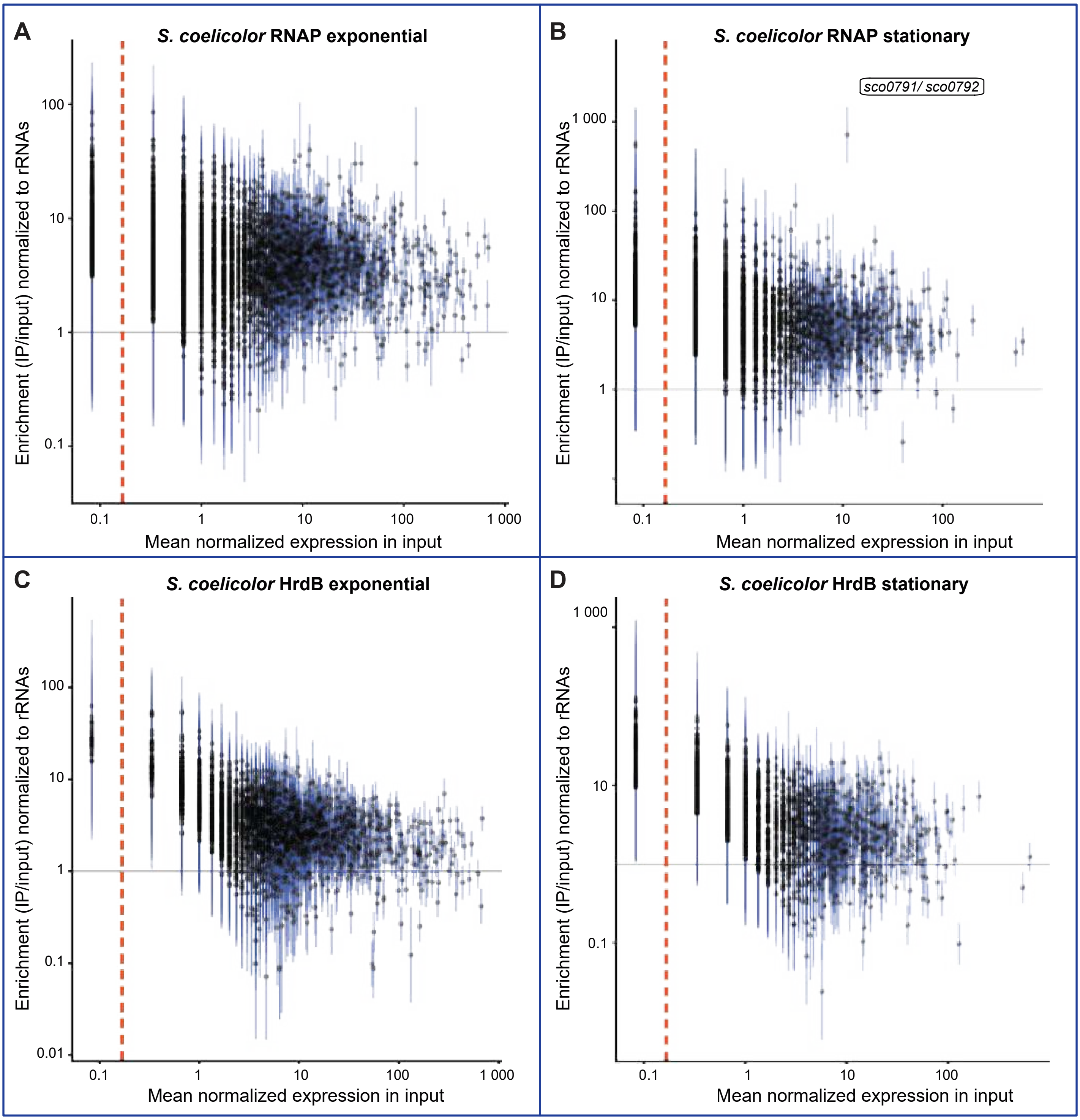

E scr0792 secondary structure prediction

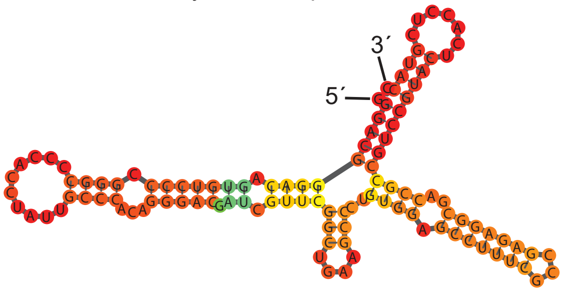

Supplementary Figure S5. Quantification of RIP-seq data in *S. coelicolor* in exponential and stationary phase for RNAP (**A, B**) and the primary σ factor, HrdB (**C, D**) for each intergenic region in the genome. For details, see legend to Figure S2. **E.** Minimum free energy secondary structure prediction of the RIP-seq enriched intergenic region transcript scr0792 generated using RNAfold (Vienna RNAfold webserver (2)). The parts of the secondary structure with the highest base pair probabilities are highlighted in red. The genome coordinates of the RIP-seq enriched fragment are NC_003888: 838,753-838,866.

Supplementary Figure S6

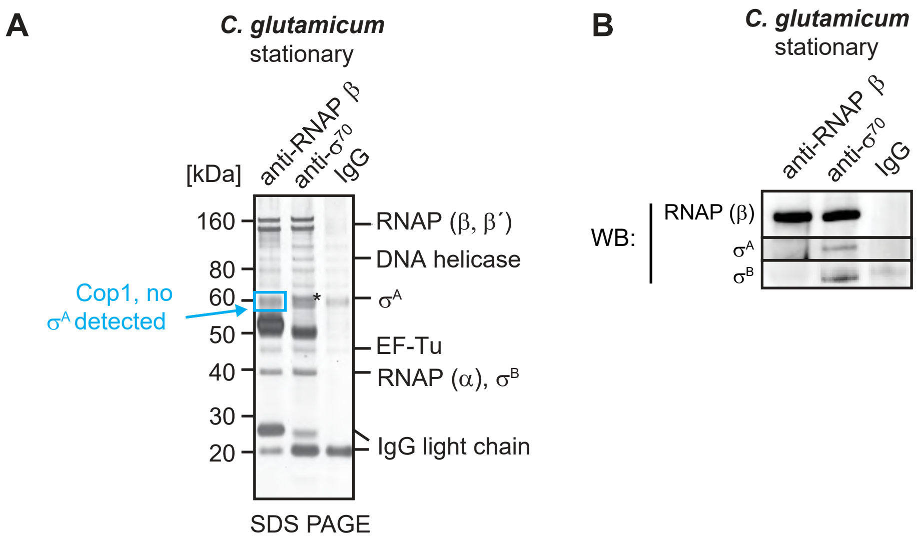

C

```
Sigma B_Cglutamicum        ------------------------------------------------------------   0
MSMEG_2758                 ----------------------------------MAATKASPATEE----------PVK-   15
Sigma A_Cglutamicum        MESSMVENNVAKKTVAKKTARKTARKAAPRVATPLGVASESPISATPARSIDGTSTPVEA   60


Sigma B_Cglutamicum        ------------------------------------------------------------   0
MSMEG_2758                 -------RTATKTPAKKAPAKRAAKSAAAKAGGKAPAKKAPAKRAAKGTAAKPEDGVTDD   68
Sigma A_Cglutamicum        ADTIETTAPAAKAPAAKAPAKKVAKK----TARKAPAKKTVAKKATTAKA-APATAKDEN   115


Sigma B_Cglutamicum        ----------------------------------------------------------MT   2
MSMEG_2758                 LEVTDDLEAEPGEDLDVEDTDLELDDLDSDDDTAVEDEEEEADAATPAVATAKAADDDID   128
Sigma A_Cglutamicum        APVDDD-------------EENLAQDEQDFDGDDFVDGIEDEED---------EDGVEALG   154
                                                                                     :

Sigma B_Cglutamicum        APSTQDLATTER------EVDPGSRRGQTNDNPSQDLVRVYLNGIGKTALLTAEDEVELA   56
MSMEG_2758                 EPSEKDKASGDFVWDEEESEALRQARKDAELTASADSVRAYLKQIGKVALLNAEEEVELA   188
Sigma A_Cglutamicum        EESEDDEEDGSSVWDEDESATLRQARKDAELTASADSVRAYLKQIGKVALLNAEQEVSLA   214
                             * .*    .       .    . * ::: . * * **.**: ***.***.**:**.**

Sigma B_Cglutamicum        QTIEVGLYAEHLLKNSEE---------PLTRAMKRDLKVLAKDGKKARSHLLEANLRLVV   107
MSMEG_2758                 KRIEAGLYATQKLAELAE------KGEKLPVQQRRDMQWICRDGDRAKNHLLEANLRLVV   242
Sigma A_Cglutamicum        KRIEAGLYATHRMEEMEEAFAAGDKDAKLTPAVKRDLRAIARDGRKAKNHLLEANLRLVV   274
                           : **.**** : : :  *          *    :**:: :.:** :*:.***********

Sigma B_Cglutamicum        SLAKRYTGRGMPLLDLIQEGNLGLIRAMEKFDYSKGFKFSTYATWWIRQAITRGMADQSR   167
MSMEG_2758                 SLAKRYTGRGMAFLDLIQEGNLGLIRAVEKFDYTKGYKFSTYATWWIRQAITRAMADQAR   302
Sigma A_Cglutamicum        SLAKRYTGRGMAFLDLIQEGNLGLIRAVEKFDYSKGYKFSTYATWWIRQAITRAMADQAR   334
                           *********** :**************:*****:**:****************.****:*

Sigma B_Cglutamicum        TIRLPVHLVEQVNKLSRIKRELYQHLGREATNEELAEESGIEESKIEMLLRQSRDPVSLD   227
MSMEG_2758                 TIRIPVHMVEVINKLGRIQRELLQDLGREPTPEELAKEMDITPEKVLEIQQYAREPISLD   362
Sigma A_Cglutamicum        TIRIPVHMVEVINKLGRIQRELLQELGREPTPQELSKEMDISEEKVLEIQQYAREPISLD   394
                           ***:***:** :***.**:*** *.**** * :**::* .*  .*:  : : :*:*:***

Sigma B_Cglutamicum        MPVGADEEAPLGDFIEDSEATDAESAVVASMRHSDIRAVLNTLEPREQDVIRLRYGLDDG   287
MSMEG_2758                 QTIGDEGDSQLGDFIEDSEAVVAVDAVSFTLLQDQLQSVLETLSEREAGVVRLRFGLTDG   422
Sigma A_Cglutamicum        QTIGDEGDSQLGDFIEDSEAVVAVDAVSFTLLQDQLQDVLETLSEREAGVVKLRFGLTDG   454
                             :* : :: **********. * .**  :: :.::: **:**. ** .*::**:** **

Sigma B_Cglutamicum        VPRTLDQIGRRFGLSRERVRQIEREVMSKLRDGERASRLREYAQ  331
MSMEG_2758                 QPRTLDEIGQVYGVTRERIRQIESKTMSKLRHPSRSQVLRDYLD  466
Sigma A_Cglutamicum        MPRTLDEIGQVYGVTRERIRQIESKTMSKLRHPSRSQVLRDYLD  498
                            *****:**: :*::***:**** :.*****. .*:. **:* :
```

LQEMGREPTPEELAER *E. coli* peptide used for antibody production

Supplementary Figure S6. A. Immunoprecipitations were performed with anti-σ^{70} and anti-RNAP antibodies and nonspecific IgG as a negative control with the lysates from exponential phase (OD_{600} ~1) and early stationary phase (OD_{600} >7, ~26 h of cultivation) *C. glutamicum* cultures, resolved on SDS-PAGE and stained with silver. The identities of protein bands were confirmed by mass spectrometry. We did not detect any σ^A in the anti-RNAP immunoprecipitation by mass spectrometry, the area of the gel used for analysis is indicated in blue. **B.** Western blot of proteins immunoprecipitated with anti-RNAP and anti-σ^{70} antibodies in *C. glutamicum*. **C.** Amino acid sequences of σ^A and σ^B from *C. glutamicum*. σ^A (*MSMEG_2758*) from *M. smegmatis* is also shown.

Supplementary Figure S7

Gene transcripts

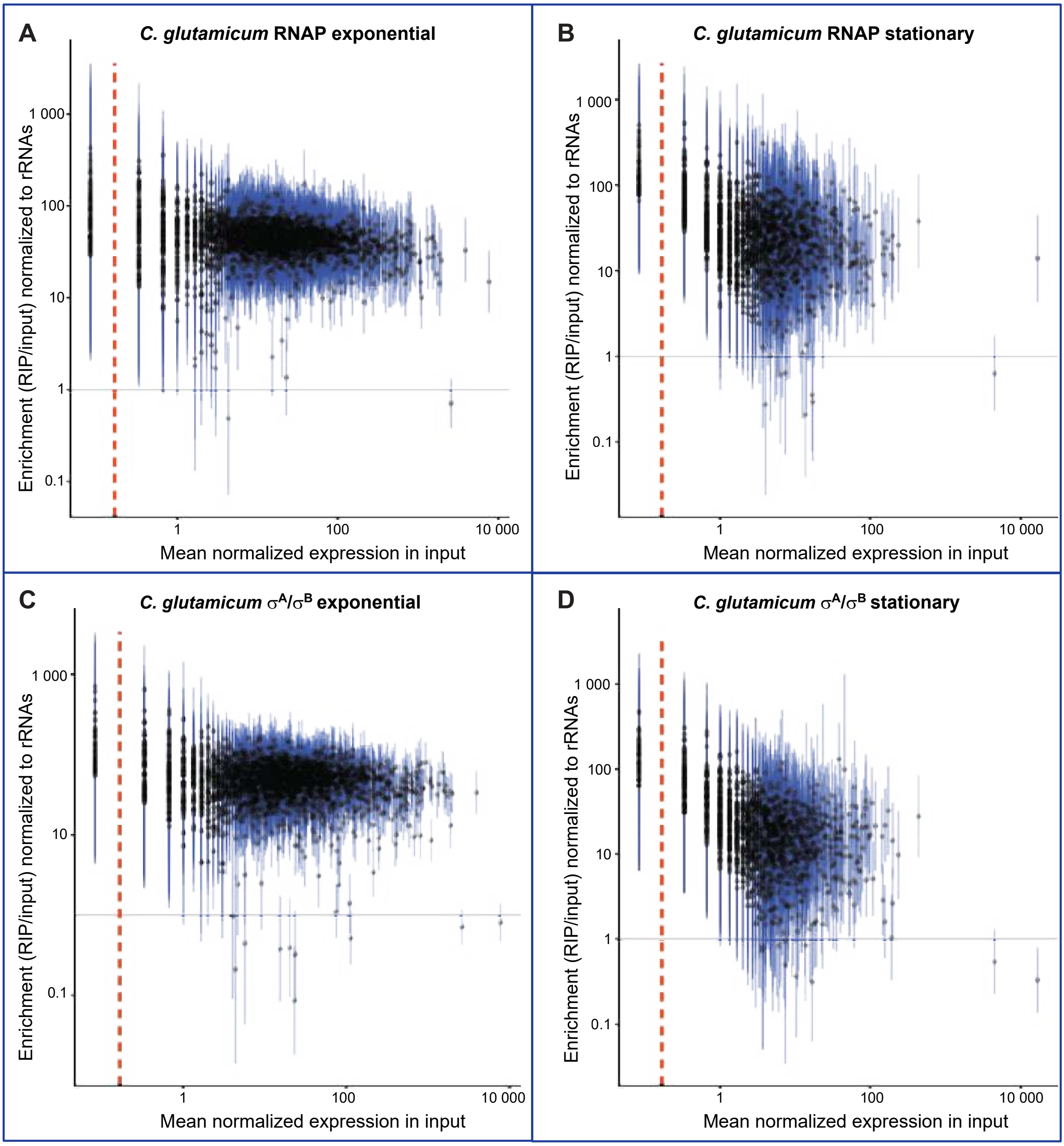

Supplementary Figure S7. Quantification of RIP-seq data in *C. glutamicum* for RNAP (**A, B**) and σ^A/σ^B (**C, D**) for each annotated gene in exponential and stationary phase. For intergenic regions, please see Figure 8. For further details, see legend to Figure S2.

Supplementary Figure S8

A

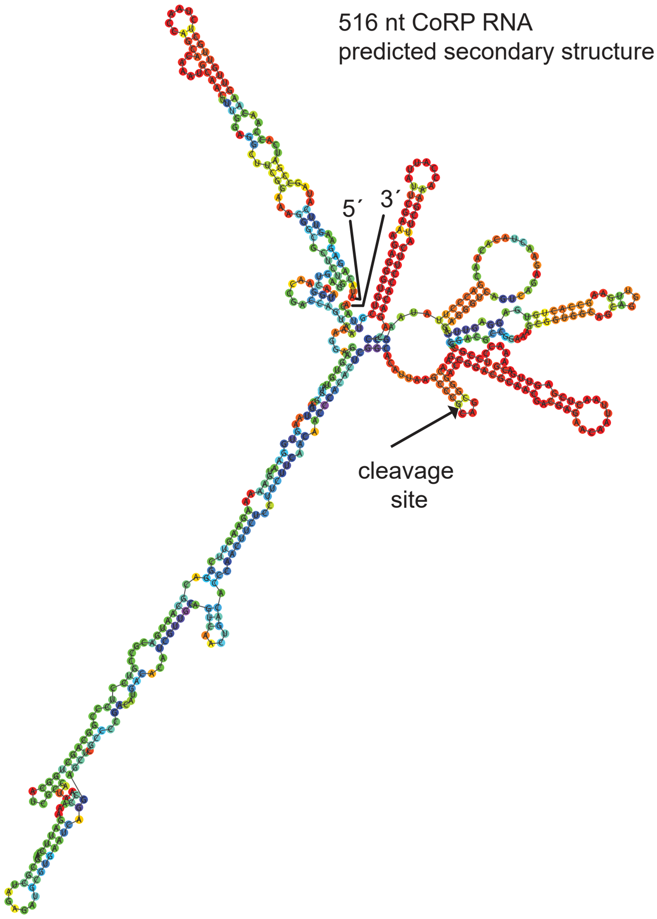

B

516 nt CoRP RNA sequence
198 nt 3´end fragment in purple

ATACAGAGAAGTTCATAGCCGATCACCAACAAGTTGTTGCTCTAACCAGCACAAATGCAACTTTGGAGGCTTCGGAAAGGGCGCTCTGGA
GTCAATGGAACCGACCAGTAAAAGGAGAGTGTATGGCAGTAAGTGGAATGAAAAAGAAGTTCGGACGCAATGACGCCGTCCTCCCGGCAG
CTGGCATCGCCATAACCAAAGATTCCAAGCGCTAGAGATGCGTGAATCAGGAGCTCGCCCCGACATGACACATCGTTGCAGTCAACTGAC
ACCCAACTTCTCCTTCTTCAACAACCCACACTCGGCACATTAAGCCCGCAGCGGGCAACAGCGGACGCAACGACGAGAACAATTAACTCG
AGTTGACGAAACCTCCGCACCGACGCCGGAAAGCGGTGGCAGCAGGTTGAAGCCACTGTGAGGAGTTGAGAGGGTCAGTCAGAGAACTAC
ACAACGACCCTTATAAGCCCAGACACCTTCATTCGAAACCATTATTCGAAAGAGGGTGTCGTTACA

Supplementary Figure S8. A. Minimum free energy secondary structure predicted for the 516 nt CoRP RNA transcript using RNAfold (Vienna RNAfold webserver (2)). The parts of the secondary structure with the highest base pair probabilities are highlighted in red. The cleavage site is marked by arrow. The genome coordinates of CoRP RNA are NC_003450.3:1,366,239-1,366,719. **B.** The CoRP RNA sequence, 198 nt fragment is in purple.

Supplementary Figure S9

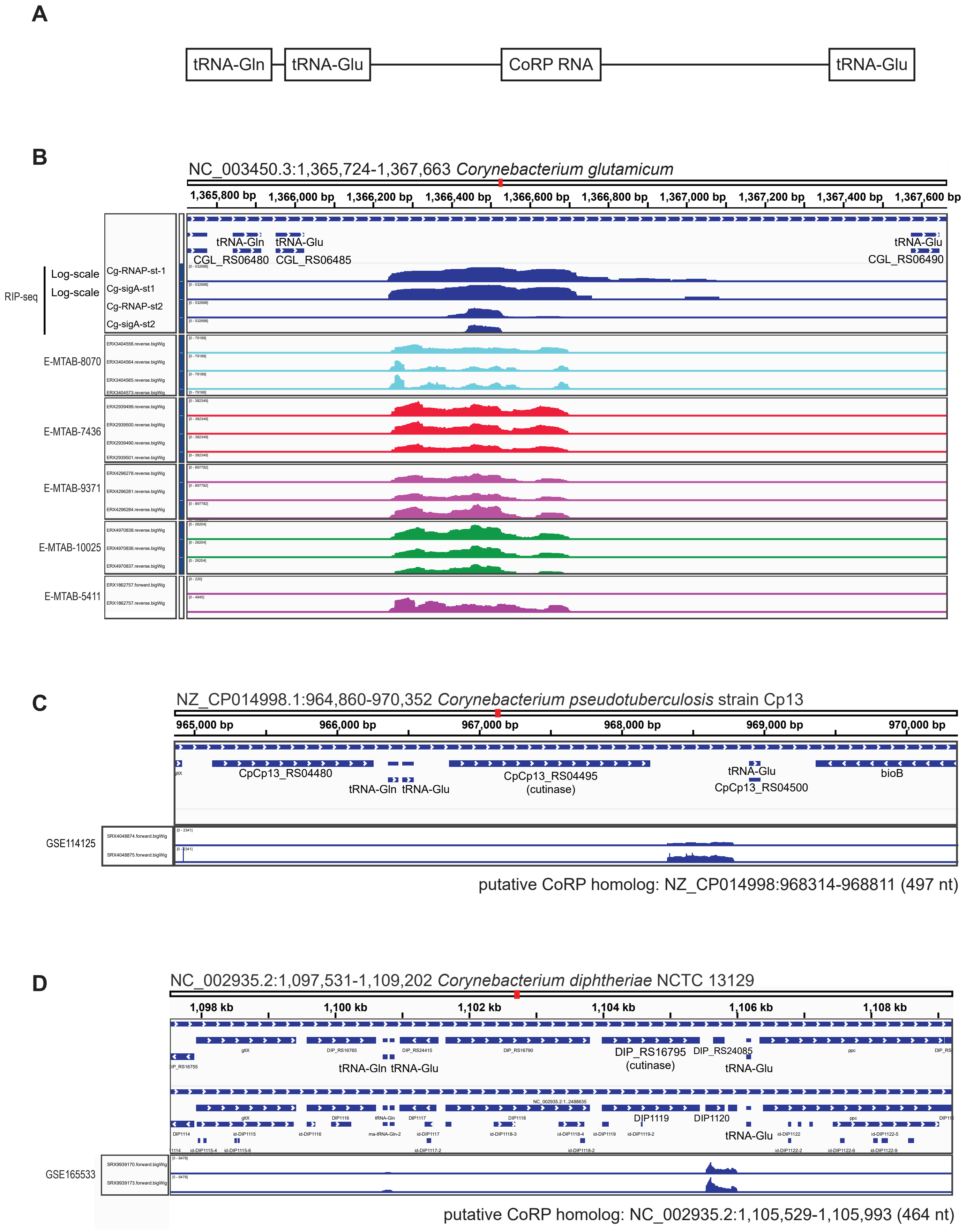

Supplementary Figure S9. A. CoRP homologs have conserved synteny and the general pattern was as follows: tRNA Gln – tRNA Glu – CoRP – tRNA Glu **B.** Datasets from various RNA-seq experiments mapped to the *C. glutamicum* genome confirm CoRP RNA expression. **C.** Datasets from *ciuA* iron-acquisition-deficient *C. pseudotuberculosis* Cp13 mutant RNA-seq experiment (3) show transcript of putative CoRP RNA homolog. **D.** Datasets from two replicas of RNA-seq experiments from wild-type *C. diphtheriae* (4) show transcript of putative CoRP RNA homolog. This transcript overlaps with the hypothetical protein DIP1120 and DIPRS24085 in two different genome annotations. In both *C. pseudotuberculosis* and *C. diphtheriae*, gene synteny of CoRP RNA homologs is conserved (5´end flanking gene cutinase, 3´end flanking gene tRNA-Glu). The nf-core/fetchngs (5) and nf-core/rnaseq (6) (with HISAT2 mapping) pipelines were used for this exploration.

Supplementary Figure S10

Intergenic region transcripts

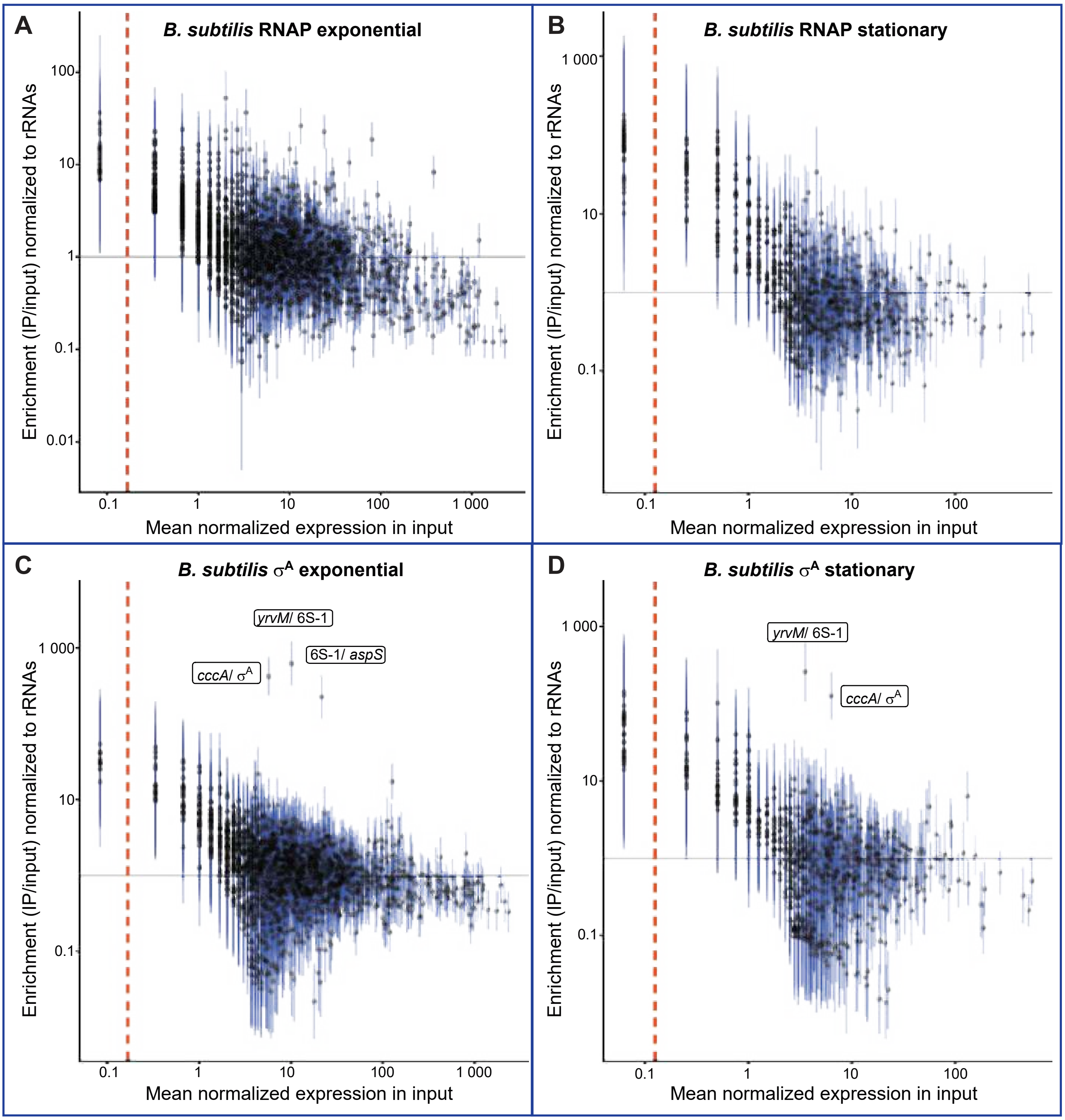

Supplementary Figure S10. Quantification of RIP-seq data in *B. subtilis* in exponential and stationary phase for RNAP (**A, B**) and σ^A (**C, D**) for each intergenic region in the genome. For further details, see legend to Figure S2.
